# Propylsulfonic Acid-Functionalized Mesostructured Natural Rubber/Silica Nanocomposites as Promising Hydrophobic Solid Catalysts for Alkyl Levulinate Synthesis

**DOI:** 10.3390/nano12040604

**Published:** 2022-02-11

**Authors:** Supphathee Chaowamalee, Ning Yan, Chawalit Ngamcharussrivichai

**Affiliations:** 1 Department of Chemical Technology, Faculty of Science, Chulalongkorn University, Bangkok 10330, Thailand; sup_7788@hotmail.com; 2 Center of Excellence on Petrochemical and Materials Technology (PETROMAT), Chulalongkorn University, Bangkok 10330, Thailand; 3 Department of Chemical and Biomolecular Engineering, National University of Singapore, 4 Engineering Drive 4, Singapore 117585, Singapore; ning.yan@nus.edu.sg; 4 Center of Excellence in Catalysis for Bioenergy and Renewable Chemicals (CBRC), Faculty of Science, Chulalongkorn University, Bangkok 10330, Thailand

**Keywords:** nanocomposite, mesoporous silica, natural rubber, in situ sol-gel, solid acid catalyst, esterification

## Abstract

Organosulfonic acid-functionalized mesoporous silica is a class of heterogeneous acid catalysts used in esterification processes due to its high surface area, shape-selective properties, and strongly acidic sites. Since water is generated as a by-product of esterification, the surface of mesostructured silica is modified to enhance hydrophobicity and catalytic performance. In this study, a series of propylsulfonic acid-functionalized nanocomposites based on natural rubber and hexagonal mesoporous silica (NRHMS-SO_3_H) with different acidities were prepared via an in situ sol-gel process using tetraethyl orthosilicate as the silica source, dodecylamine as the nonionic templating agent, and (3-mercaptopropyl)trimethoxysilane as the acid-functional group precursor. Compared with conventional propylsulfonic acid-functionalized hexagonal mesoporous silica (HMS-SO_3_H), NRHMS-SO_3_H provided higher hydrophobicity, while retaining mesoporosity and high surface area. The catalytic activity of synthesized solid acids was then evaluated via batch esterification of levulinic acid (LA) with alcohols (ethanol, *n*-propanol, and *n*-butanol) to produce alkyl levulinate esters. NRHMS-SO_3_H exhibited higher catalytic activity than HMS-SO_3_H and ultra-stable Y (HUSY) zeolite owing to the synergistic effect between the strongly acidic-functional group and surface hydrophobicity. The activation energy of the reaction over the NRHMS-SO_3_H surface was lower than that of HUSY and HMS-SO_3_H, suggesting that tuning the hydrophobicity and acidity on a nanocomposite surface is a compelling strategy for energy reduction to promote catalysis.

## 1. Introduction

Levulinic acid (LA), 4-oxo pentanoic acid, or *γ*-ketovaleric acid is a versatile biomass-derived C5 chemical platform that has attracted vast attention in the synthesis of renewable chemicals, fuels, lubricants, and polymers. LA is a short-chain fatty acid molecule (C_5_H_8_O_3_), possessing a ketone carbonyl group (C = O) and an acidic carboxylic group (COOH) [1,2,3,4], which interacts with different functional groups to form various derivatives, making it an ideal chemical building block [5]. Alkyl levulinate esters (ALE), commonly produced by acid-catalyzed LA esterification with simple alcohols, namely primarily ethanol and *n*-butanol, belong to a class of bio-based chemicals with numerous applications, such as green solvents, latex coatings, flavorings, and fragrance [6]. ALE also forms a viable petroleum-diesel-miscible fuel component, preventing fossil fuel depletion and carbon dioxide emission. These esters exhibit exceptional characteristics, including good energy content, high lubricity, and sulfur-free composition. In addition, ALE possesses properties that resemble those of fatty acid methyl esters, but with better fluidity under low-temperature conditions and flash point stability [7,8,9].

Esterification is commercially performed in the liquid phase using homogeneous catalysts, such as sulfuric acid and *p*-toluenesulfonic acid [10,11]. Even though these soluble acids provide a high yield, high conversion, and short reaction time, there are several drawbacks including equipment corrosion, catalyst separation, product quality, catalyst toxicity, and waste generation. Heterogeneous catalysis exhibits superior characteristics over homogenous catalysis due to their simplified separation, recyclability, and environmental friendliness, thus attracting immense interest for esterification [12]. However, the conventional solid acid catalysts, including zeolites and ion-exchange resins, have some limitations in terms of their texture and thermal stability [13]. Ogino et al. [14] developed a series of sulfonic acid-containing carbons derived from phenolic resins with tunable pore sizes and surface chemical functionalities. The hydrophobicity and substantial pore size enhanced their catalytic performances in an acid-catalyzed liquid phase reaction. Furthermore, by precisely tuning the sulfonic acid group density, the activity of carbon-based catalysts was improved in LA esterification with ethanol due to hydrogen-bonding between the functional group on the catalyst surface and the *γ*-keto group of LA [15].

Propylsulfonic acid-functionalized mesoporous silica (HMS-SO_3_H) is a heterogeneous acid catalyst, promising for esterification due to its high surface area, shape-selectivity, and strongly acidic moieties [16]. The propylsulfonic group precursor (3-mercaptopropyl) trimethoxysilane (MPTMS) can be anchored on the mesoporous silica surface via post-grafting or the co-condensation method. The direct synthesis of functionalized mesoporous silica via sol-gel chemistry, wherein the silica source is co-condensed with MPTMS [17], provides better loading and distribution of functional groups than the post-grafting approach via the silylation reaction [18,19,20]. However, pure silica-based mesostructured catalysts show high affinity to water due to the high content of surface silanol groups (Si-OH), retarding the free fatty acid esterification [21]. This drawback greatly limits the ester production since esterification is a reversible reaction liberating water as the by-product, which further promotes the hydrolysis of esters back into the reactants, thus lowering the conversion [22,23]. To overcome this problem, the materials are made hydrophobic to continuously remove water from the porous system. Therefore, mesostructured polymer/silica nanocomposites with enhanced hydrophobicity have been proposed using natural rubber as a hydrophobicity-enhancing agent [24]. Nuntang et al. [25] successfully synthesized propylsulfonic acid-functionalized mesoporous natural rubber/silica nanocomposites (NRHMS-SO_3_H) through the in situ sol-gel method and co-condensation with MPTMS. The synthesized materials acted as solid acid catalysts in the esterification of carboxylic acid with ethanol without severe activity loss in the presence of water.

Herein, we explored the synthesis of NRHMS-SO_3_H nanocomposites with different loading levels of MPTMS via a one-pot approach wherein MPTMS was co-condensed with a silicate framework during the formation of natural rubber (NR)/silica nanocomposite via in situ sol-gel chemistry. Several characterization techniques examined the synthesized materials for their physicochemical properties. The catalytic performance of the NRHMS-SO_3_H nanocomposite was compared with those of various solid acidic materials in the esterification of LA with alcohols (ethanol, *n*-propanol, and *n*-butanol). Kinetic model analysis and the activation energy of LA esterification were also evaluated to provide information for further simulation and optimization [26,27]. The results suggested that NRHMS-SO_3_H nanocomposites retained their mesoporosity and physicochemical properties while providing higher catalytic activity for levulinic acid esterification compared with HUSY zeolite and conventional HMS-SO_3_H.

## 2. Materials and Chemical Reagents

Tetraethyl orthosilicate (TEOS) (AR grade, 98%), dodecylamine (DDA) (AR grade, 98%), (3-mercaptopropyl)trimethoxysilane (MPTMS) (AR grade, 95%), LA (AR grade, 98%), *N*-methyl-*N*-(trimethylsilyl) trifluoroactamide (MSTFA) (Synthesis grade, 98.5%), and methyl undecanoate (C11) (GC grade, 99%) were purchased from Sigma Aldrich (St. Louis, MO, USA). Sulfuric acid (H_2_SO_4_) (AR grade, 98%), tetrahydrofuran (THF) (AR grade, 99.5%), hydrogen peroxide (H_2_O_2_) (AR grade, 30%), absolute ethanol (AR grade, 99.9%), *n*-propanol, and *n*-butanol (Both AR grade, 99.5%) were obtained from QRëC (Chonburi, Thailand). Ethanol (commercial grade, 95%) was purchased from Alcoh (Bangkok, Thailand). NR (Standard Thai Rubber grade 5L) was supplied by Thai Hua Chumporn Natural Rubber Co. Ltd. (Bangkok, Thailand). 1, 4-Dioxane (AR grade, 99%) was obtained from Thermo Fisher (Fair Lawn, NJ, USA) and sodium sulfate anhydrous (Na_2_SO_4_) was acquired from Carlo Erba (Val de Reuil, France). All chemical reagents and materials were used without further purification.

### 2.1. Synthesis of HMS-SO_3_H Materials

Propylsulfonic acid-functionalized hexagonal mesoporous silica was synthesized via the sol-gel method and functionalized with MPTMS via co-condensation, as mentioned elsewhere [24]. Typically, 3.75 g of DDA was dissolved in THF (26.67 g) under stirring for 30 min. To this solution, deionized (DI) water (53.05 g) was added, and the resulting mixture was stirred for another 30 min. Then, 10.5 g of TEOS was added dropwise, followed by stirring at 40 °C for 30 min. MPTMS and H_2_O_2_ solutions, at a molar ratio of 1:7, were slowly added and the mixture was stirred at 40 °C for 1 h. After aging the resulting mixture at 40 °C for 1 d, the solid product was recovered by filtration and dried at 60 °C for 18 h. Template removal was performed by extraction with 0.05 M H_2_SO_4_/ethanol at 80 °C for 4 h, and the resulting solid was thoroughly washed with ethanol and dried at 60 °C for 12 h. The synthesized material was called HMS-SO_3_H (x), where x indicates the MPTMS:TEOS molar ratio used in the synthesis mixture.

### 2.2. Synthesis of NRHMS-SO_3_H Materials

The acidic NR/HMS nanocomposites were prepared by the in situ sol-gel method, which was modified from a previous procedure [24]. First, 0.5 g of the NR sheet was directly swollen in TEOS (10.5 g) overnight. Around 2 g of TEOS was absorbed into the NR sheet. The obtained NR gel was then stirred overnight in THF (26.67 g). Then, 3.75 g of DDA was slowly added to the resulting colloidal mixture and stirred for another 30 min, followed by the addition of TEOS (8.5 g) under stirring. After 30 min, deionized (DI) water (53.05 g) was added dropwise and the resulting mixture was stirred at 40 °C for 30 min. To the mixture, MPTMS and an H_2_O_2_ solution (the same MPTMS:H_2_O_2_ molar ratio as in HMS-SO_3_H synthesis) were slowly added, followed by stirring at 40 °C for 1 h. The mixture was then aged at 40 °C for 2 d. The solid product was recovered by precipitation in ethanol, filtered, and dried at 60 °C for 18 h. The template extraction and product finishing were performed in the same manner as the HMS-SO_3_H synthesis. The synthesized material was called NRHMS-SO_3_H (x), where x indicates the MPTMS:TEOS molar ratio used in the synthesis mixture.

### 2.3. Characterization of Synthesized Materials

Powder X-ray diffraction (XRD) was applied to access the mesostructure ordering of materials obtained using a Bruker D8 Advanced X-ray diffractometer equipped with Cu Kα radiation operated at an X-ray power of 40 kV and 40 mA. The XRD patterns were recorded at room temperature, scanning from a 2θ of 1–10° at a 0.02° step size and 1 s count time. The hexagonal lattice parameter (a_0_) was calculated from the interplanar spacing (d-spacing) of the (100) reflection peak using the equation: a_0_ = 2d_100_/√3.

Nitrogen (N_2_) adsorption–desorption measurement at −196 °C was performed on a Mircrometrics ASAP2020 surface area and porosity analyzer to determine the textural properties of the synthesized materials. All samples were degassed at 150 °C for 2 h prior to the measurements. The specific surface area (S_BET_) was calculated from the adsorption data in the relative pressure (P/P_0_) range of 0.02−0.2 using the Brunauer–Emmett–Teller (BET) equation. External surface area (S_ext_) was estimated from the t-plot slope. The mesopore volume (V_P_) was calculated from the intercept of the linear portion of the t-plot in the relative pressure range, above which N_2_ was condensed inside the primary mesopores. The pore diameter (D_p_) was determined by the Barrett–Joyner–Halenda (BJH) calculation using the desorption data. The total pore volume (V_T_) was attained from the cumulative N_2_ adsorbed volume at P/P_0_ of 0.990.

Thermogravimetric analysis (TGA) was used to determine the silica and rubber content of the nanocomposite catalysts. Each sample (~10 mg) was heated from 40–850 °C at a rate of 10 °C/min under an air flow (50 mL/min) using the PerkinElmer Pyris Diamond thermogravimetric analyzer.

Functional groups of the synthesized materials were revealed via attenuated total reflection Fourier-transform infrared spectroscopy (ATR-FTIR). ATR-FTIR spectra were recorded on the Nicolet iS10 FT-IR spectrometer over 500–4000 cm^−1^ with 64 scans at a resolution of 4 cm^−1^.

The mesostructured arrangement of materials was visualized by transmission electron microscopy (TEM) using the JEOL JEM-2010 transmission electron microscope at an accelerating voltage of 200 kV. The distribution of the main elements in the synthesized materials was examined via scanning TEM with energy dispersive X-ray spectroscopy (STEM-EDS) mapping using the JEOL JEM-2010 transmission electron microscope at an accelerating voltage of 200 kV in the dark field mode.

The sulfur incorporated into the functionalized materials was measured using the PE 2400 CHNS/O Elemental analyzer. Cystine was used as the standard for eight repetitive analyses to obtain the K factor. Approximately, 2 mg of the standards and samples was used to analyze and correct the data with K factor.

The chemical states of oxygen and sulfur on the material surface were analyzed by X-ray photoelectron spectroscopy (XPS) using the Kratos Axis Ultra DLD X-ray photoelectron spectrometer equipped with a monochromic Al Kα X-ray source (1486.7 eV) operated at 15 kV and 5 mA. Survey scans were measured at a spot size of 400 μm and a constant pass energy of 200 eV. The calibration was performed by setting the C1s band at 284.5 eV. The deconvolution of high-resolution XPS element spectra was performed using the XPSPEAK41 software.

The acidity of functionalized materials was evaluated by acid–base titration. Generally, 0.5 g of sample was mixed with 10 mL of THF and 10 mL of ethanol. The mixture was shaken at room temperature for 24 h. The resulting mixture was then titrated with 0.02 M NaOH aqueous solution.

Hydrophobicity of the synthesized materials was determined by H_2_O adsorption-desorption measurement using the BEL Japan BELSORP-max instrument. The sample was pretreated at 150 °C for 2 h under vacuum, and the measurement was performed at room temperature. The H_2_O monolayer adsorbed volume (V_m, H2O_) was determined using the adsorption data within the P/P_0_ range of 0.2.

### 2.4. Catalytic Esterification of LA with Alcohols

Esterification of LA with alcohols was performed with HUSY, HMS-SO_3_H, and NRHMS-SO_3_H catalysts. In this study, three types of alcohols (ethanol, *n*-propanol, and *n*-butanol) were esterified with LA to observe the effect of alcohol structure on the activity of catalysts. The reaction was conducted batchwise in a 50-mL three-neck round bottom flask equipped with a magnetic stirrer and a reflux system. The reaction temperature was controlled by a silicone oil bath equipped with a digital thermocouple. In each experiment, LA and alcohol (molar ratio = 1:5) were homogeneously mixed in the flask. Subsequently, the catalyst pretreated at 100 °C for 2 h was added to the reaction solution. The typical reaction condition was 2.5 wt.% catalyst loading (based on the LA weight). A certain amount of reaction mixture was withdrawn at different time intervals up to 5 h of reaction time and subjected to composition analysis.

The reaction product composition was evaluated by the Agilent 7890A gas chromatograph equipped with a DB-5ht capillary column and flame ionization detector. Prior to analysis, water was eliminated from the reaction product by adding Na_2_SO_4_. The yield of the ester product was quantified using the internal standardization method. C11 was used as a reference. MSTFA was used to convert the remaining LA and alcohol into nonpolar derivatives. The sample volume was finally made by adding 1, 4-dioxane.

## 3. Results and Discussion

### 3.1. Physicochemical Properties of HMS-SO_3_H and NRHMS-SO_3_H

**Figure 1:**
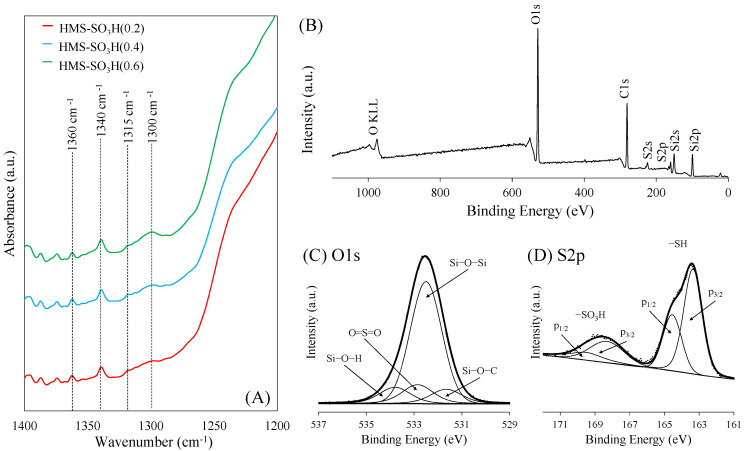
Representative (**A**) ATR-FTIR spectra of HMS-SO_3_H with different MPTMS loading levels. (**B**) Wide-scan XPS spectrum, and core-level, high-resolution (**C**) O1s and (**D**) S2p XPS spectra of HMS-SO_3_H (0.4). The wide-scan and core-level high-resolution XPS spectra of other NRHMS-SO_3_H samples are shown in Figures S2–S7 (SM).

HMS-SO_3_H functionalization with different MPTMS loading levels was validated through the ATR-FTIR and XPS spectra, as shown in Figure 1. The wide-scan XPS spectrum of HMS-SO_3_H (0.4) confirmed the presence of sulfur and silica framework structures, exhibiting six characteristic bands at 284.5, 533, 233, 160, 151, and 100 eV, corresponding to C1s, O1s, S2s, S2p, Si2s, and Si2p, respectively. The chemical state of oxygen exhibited the siloxane bond (Si−O−Si) as the material core structure at 532.6 eV. Other chemical states were also seen at 531.8 and 533.9 eV, corresponding to Si−O−C of remnant ethoxy groups and Si−O−H of silanol groups, respectively [28]. In-depth ATR-FTIR spectra of the synthesized samples are illustrated in the Supplementary Material (SM) Figure S1. The bands related to sulfonic acid groups were observed in doublets at 1340 cm^−1^ and 1315 cm^−1^, attributed to the stretching vibration of –SO_3_ species [29]. The band at 1360 cm^−1^ corresponded to the S = O stretching [24]. The small band at 1300 cm^−1^ indicated the asymmetric stretching vibration of S = O [30]. The high resolution O1s XPS spectrum affirmed the chemical state of sulfur as O = S=O moieties of sulfonic acid groups, detected at 533.0 eV [31]. Additionally, the bands corresponding to sulfonic acid species were detected from a high-resolution S2p spectrum at 166.2–170.5 eV (deconvoluted into two orbital splitting states, 2Sp1/2 at 168.0 eV and 2Sp3/2 at 169.2 eV) [32,33]. However, the chemical state survey of sulfur displayed the presence of a thiol group, located between 161.8 eV and 166.2 eV (deconvoluted into two orbital splitting states, S2p1/2 at 163.6 eV and 2Sp3/2 at 164.8 eV). This unoxidized thiol group was not observed in the ATR-FTIR spectra due to the weak signal of S−H species [34]. Notably, thiol group oxidation strongly relies on temperature [35]; hence, the total oxidation of the thiol group could not occur at 40 °C. Besides, thiol groups were possibly located within the sublayer of the silica framework, thus remaining in unoxidized forms [36].

**Figure 2:**
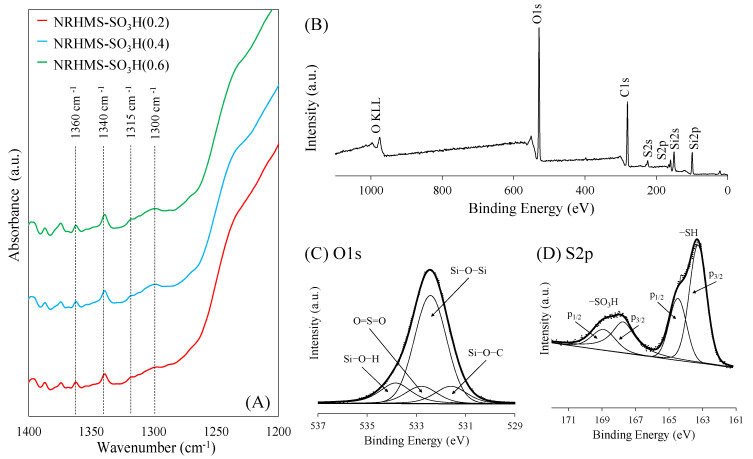
Representative (**A**) ATR-FTIR spectra of NRHMS-SO_3_H with different MPTMS loading levels. (**B**) Wide-scan XPS spectrum, and core-level, high-resolution (**C**) O1s and (**D**) S2p XPS spectra of NRHMS-SO_3_H (0.4). The wide-scan and core-level high-resolution XPS spectra of other NRHMS-SO_3_H samples are shown in Figures S8–S13 (SM).

The same spectroscopic techniques accessed the functional groups of NRHMS-SO_3_H with different loading levels of MPTMS, as shown in Figure 2. These spectra indicate that HMS-SO_3_H and NRHMS-SO_3_H exhibited similar chemical natures. The oxygen states of NRHMS-SO_3_H (0.4) nanocomposites were similar to those of HMS-SO_3_H (0.4) but different in relative concentrations (Table S1), wherein NRHMS-SO_3_H (0.4) possessed a higher amount of unhydrolyzed and uncondensed silica than HMS-SO_3_H (0.4). NR possibly hindered the formation of silica framework with functional groups, resulting in unhydrolyzed silica precursor and uncondensed silica species, thereby having less propensity to form a siloxane bond. As per the high-resolution S2p spectrum, the sample possessed both unoxidized and oxidized thiol species.

**Figure 3:**
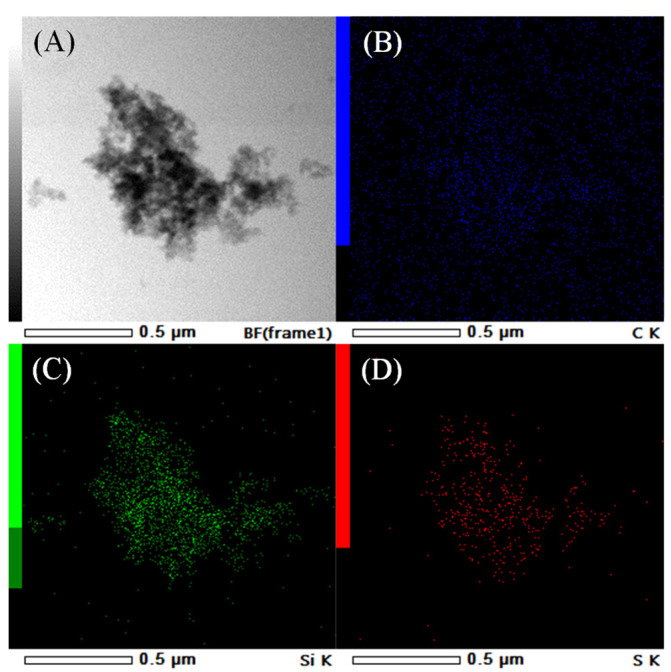
Representative (**A**) TEM of HMS-SO_3_H (0.4) at a magnification of 100,000× and EDS mapping images of elements (**B**) C, (**C**) Si, and (**D**) S.

**Figure 4:**
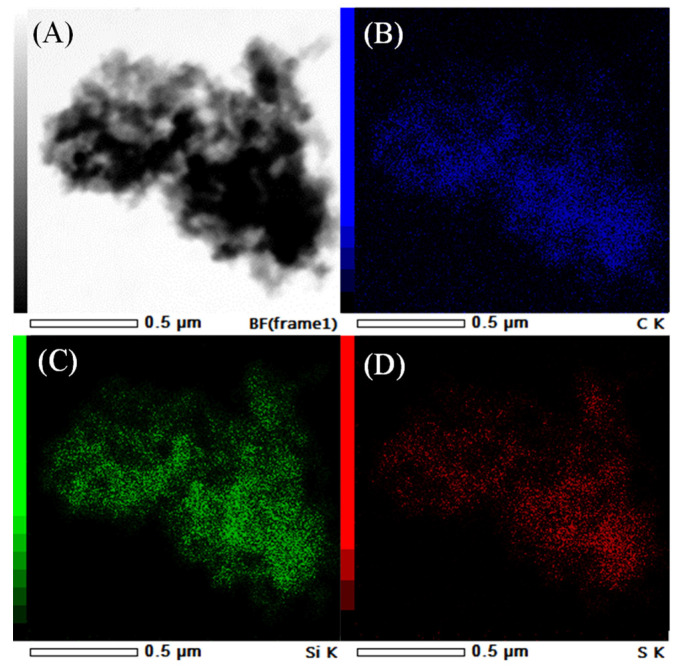
Representative (**A**) TEM of NRHMS-SO_3_H (0.4) at a magnification of 100,000× and EDS mapping images of elements (**B**) C, (**C**) Si, and (**D**) S.

The total sulfur content of the functionalized materials is summarized in Table 1. An increase in the MPTMS loading level in all cases resulted in higher sulfur content. Compared with the HMS-SO_3_H series, NRHMS-SO_3_H samples provided slightly lower sulfur content as NR hampered the formation of the silicate network, hindering the further incorporation of MPTMS into the mesostructured framework of the nanocomposite [24]. STEM-EDS was later applied to investigate the dispersion of sulfur species in the materials. As shown in Figure 3 and Figure 4, sulfur was homogeneously distributed over the material particles for both samples, confirming that functionalization via co-condensation provided a uniform distribution of organic moieties even with NR.

**Figure 5:**
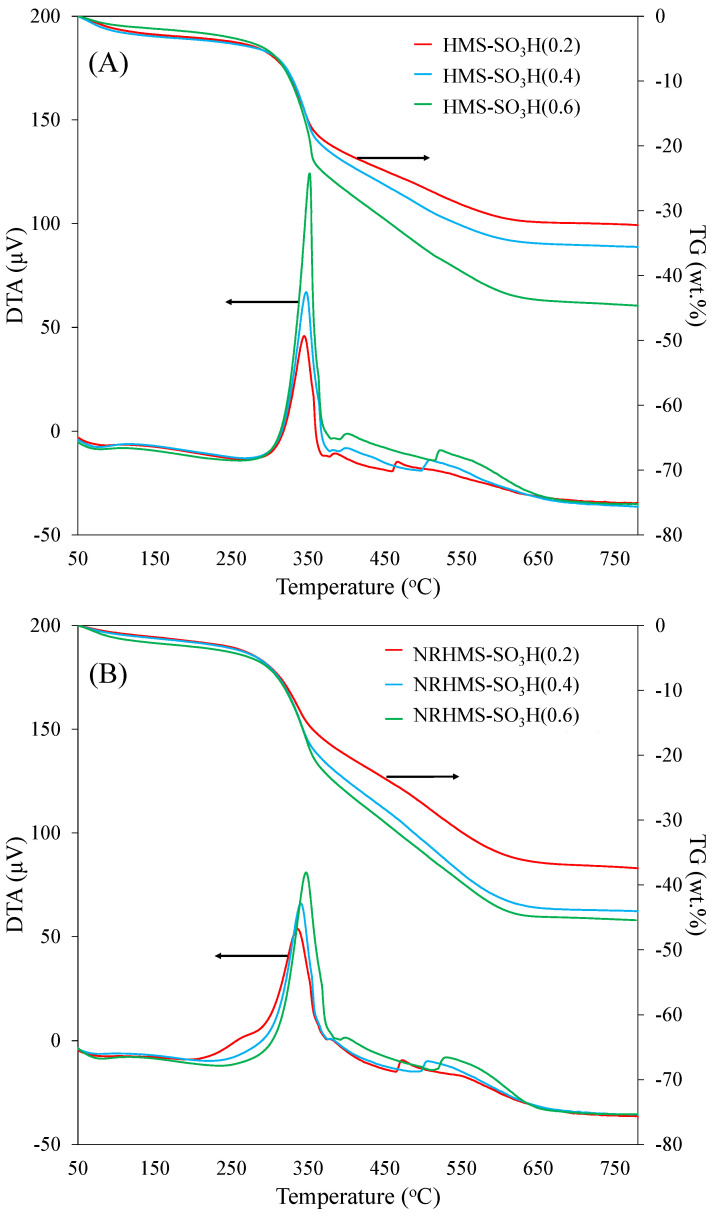
Representative TG and DTA curves of (**A**) HMS-SO_3_H and (**B**) NRHMS-SO_3_H materials with different MPTMS loading levels.

The TG and DTA curves of the HMS-SO_3_H and NRHMS-SO_3_H series are compared in Figure 5A,B, respectively. For the HMS-SO_3_H series, all samples exhibited three-step weight loss. First, a weak endothermic peak observed between 50 °C and 150 °C was attributed to the desorption of physisorbed water and ethanol from the material surface. The second instance of weight loss was located in the range of 250–450 °C corresponding to the decomposition of the mercaptopropyl group and the remnant ethoxy group [37]. The final exothermic step, in the range of 450–650 °C, was ascribed to the decomposition of alkyl sulfonic groups, the removal of carbon residue, and the dehydroxylation of silanol groups on the material surface [38]. The remaining weight of the solid was used to calculate the silica content in the materials.

The NRHMS-SO_3_H series provided similar weight loss profiles for the HMS-SO_3_H counterparts, but with a broader exothermic step at 200–450 °C due to the additional weight loss from the decomposition of the rubber phase. Hence, the exact amount of the NR component incorporated in the nanocomposites could not be determined [24]. To estimate the incorporated NR content of nanocomposites, non-functionalized NRHMS was analyzed as a representative material using TGA, showing 12 wt.% of NR content. Notably, NRHMS-SO_3_H (0.2) provided a small exothermic peak at 250 °C owing to the auto-oxidation of loosely entrapped NR molecules within the silica framework [28].

**Figure 6:**
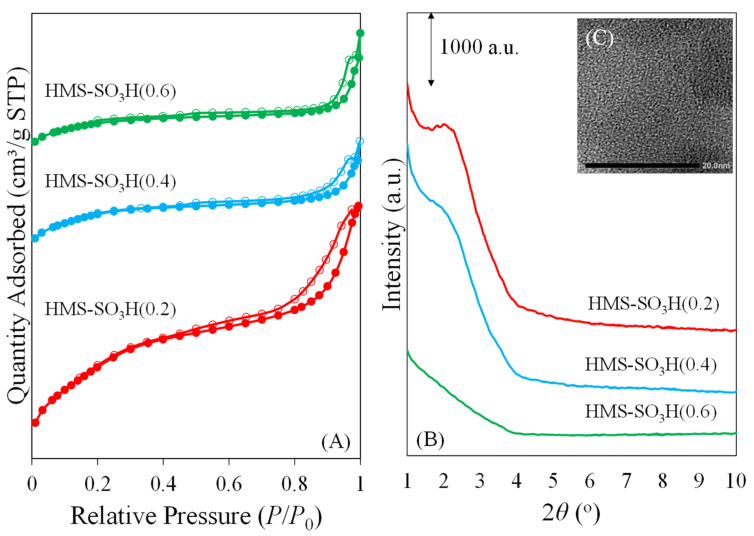
Representative (**A**) N_2_ physisorption isotherms and (**B**) low-angle XRD patterns of HMS-SO_3_H with different loading levels. (**C**) TEM images of HMS-SO_3_H (0.4) at a magnification of 300,000×.

**Figure 7:**
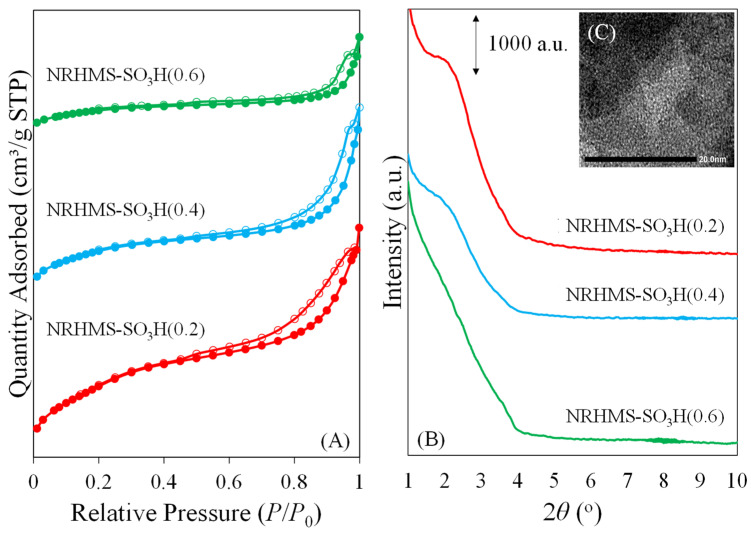
Representative (**A**) N_2_ physisorption isotherms and (**B**) low-angle XRD patterns of NRHMS-SO_3_H with different loading levels. (**C**) TEM images of NRHMS-SO_3_H (0.4) at a magnification of 300,000×.

Both HMS-SO_3_H and NRHMS-SO_3_H series provided similar N_2_ physisorption isotherms of type IV (Figure 6A and Figure 7A), representing a hexagonally mesostructured framework, as also confirmed via low-angle XRD patterns (Figure 6B and Figure 7B). A large hysteresis loop at P/P_0_ of 0.8–1.0 corresponded to the interparticle voids from the agglomeration of particles. A continuous reduction in the hysteresis loop was seen for the increased MPTMS loading from 0.2 to 0.4, suggesting increased chemically bonded MPTMS on the silica framework via the co-condensation method. As a result, the mesophase assembly was disturbed, shortening the long-range ordered structure formation [39]. Nevertheless, the TEM images of HMS-SO_3_H (0.4) and NRHMS-SO_3_H (0.4) showed the retention of a wormhole-like motif structure. S_BET_, V_p_, and V_T_ decreased with an increased MPTMS loading level, indicating successful functionalization on the material surface (Table 2) [40]. At the MPTMS/TEOS ratio of 0.6, the disappearance of the (100) reflection peak indicated the collapse of the mesoporous structure. It should be noted that the NRHMS-SO_3_H series provided decreased S_BET_, V_P_, and V_T_ but with a thicker mesoporous wall, indicating that the rubber phase was successfully infiltrated between the mesostructured silica frameworks [41]. Surprisingly, NRHMS-SO_3_H (0.4) exhibited a relatively small reduction in the textural properties, compared with HMS-SO_3_H (0.4). For example, S_BET_ of the NRHMS-SO_3_H series slightly decreased from 594 m^2^ g^−1^ to 400 m^2^ g^−1^ when increasing the MPTMS/TEOS ratio from 0.2 to 0.4, while HMS-SO_3_H (0.4) exhibited a 36% reduction of S_BET_ from HMS-SO_3_H (0.2). It was predicted that the presence of NR reduced the polarity of the material surface by covering a part of silanol groups, further preventing the aggregation of MPTMS that functionalized at the pore entrance of mesoporous nanocomposites.

The hydrophobicity of HMS-SO_3_H and NRHMS-SO_3_H materials was evaluated by H_2_O adsorption–desorption measurement (Table 2). Both material series exhibited an increased V_m, H2O_ with the increasing MPTMS due to the hydrophilic sulfonic acid groups that enhanced the water affinity of the material surface. The NRHMS-SO_3_H series provided lower water adsorption than the HMS-SO_3_H counterparts due to the dispersed rubber phase, creating a hydrophobic environment on the surface of nanocomposites. According to a previous study, the hydrophobicity of the material surface exhibited superior catalytic activity in the esterification reaction by preventing water absorption on the acid sites [42]. However, increased hydrophobicity affected the adsorption rate of reactants and created a trade-off effect between the hydrophobic character and physicochemical properties, such as losing acidic properties to gain hydrophobicity [43], which can be optimized by varying the loading level of organo-functional groups on the catalyst surface.

The total acidity of the HMS-SO_3_H and NRHMS-SO_3_H series was mainly evaluated via acid–base titration (Table 1). HMS-SO_3_H possessed higher acidity than NRHMS-SO_3_H at the same MPTMS loading level due to higher sulfur content. Furthermore, XPS analysis was used to quantitate the sulfonic acid groups, thus acidity, at the external surface of synthesized materials. In all cases, the acidity obtained via both techniques was lower than the theoretical acidity, calculated from the total sulfur content. This result confirmed that some thiol groups were not fully oxidized to sulfonic acid groups. The external surface acidity was comparable with the total acidity for both materials synthesized with the MPTMS/TEOS ratios of 0.2 and 0.4. It indicates a uniform distribution of sulfonic acid groups inside the primary mesopores and on the material external surface [24]. However, at an increased MPTMS loading level, the acidity obtained via the acid–base titration (0.90 mmol/g and 0.80 mmol/g for HMS-SO_3_H (0.6) and NRHMS-SO_3_H (0.6), respectively) was noticeably less than that determined by the XPS analysis (1.16 mmol/g and 1.29 mmol/g for HMS-SO_3_H (0.6) and NRHMS-SO_3_H (0.6), respectively). It was explained by the presence of some inaccessible sulfonic acid groups due to the non-uniform distribution of the functional groups at the pore entrance and on the external surface.

**Scheme 1:**
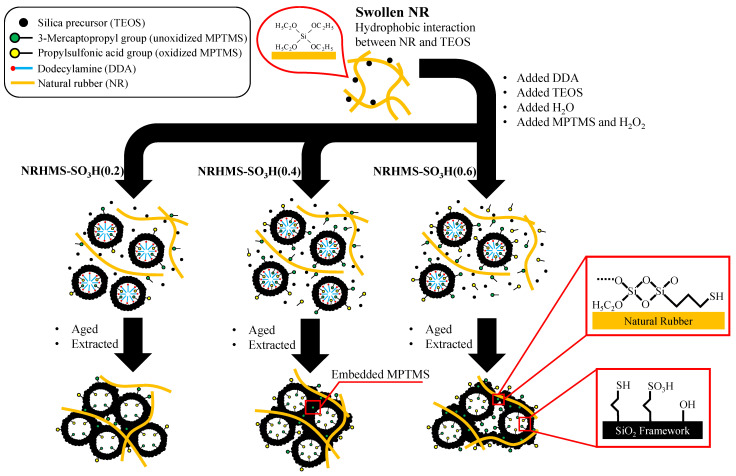
Proposed mesostructured NRHMS-SO_3_H formation.

The formation of the mesostructured NRHMS-SO_3_H material series is depicted in Scheme 1. Firstly, TEOS was infiltrated in NR via the hydrophobic interaction between the polymeric chain of NR and remnant ethoxy groups from silica precursors [44]. During the formation of the nanocomposite mesophase via cooperative self-assembly [28], the functional group precursors were uniformly distributed on the external surface within the mesoporous network and embedded in the sublayer of the silica framework. Additionally, NR induced the hydrophobic interaction between NR and silica precursors (TEOS and MPTMS), resulting in the NR incorporation in the mesoporous silica framework [45]. After H_2_O_2_ addition, thiol groups were oxidized to some extent into the propylsulfonic acid group, corresponding to a low oxidation temperature and the inaccessibility of some thiol groups. At an MPTMS/TEOS ratio of 0.2, the amount of MPTMS incorporated into the mesopores was relatively low, while the material still retained ordered mesoporosity. Hence, NRHMS-SO_3_H (0.2) exhibited an ordered mesoporous structure with higher surface area and larger mesopores, but relatively low acidity. When increasing the MPTMS loading, the number of functional groups within mesopores also increased; however, the mesophase self-assembly was partially disturbed. As a result, the hexagonal mesoporous structure became increasingly disordered and eventually collapsed at the MPTMS/TEOS ratio of 0.6. It is worth noting that the pore diameters of materials at MPTMS/TEOS ratios of 0.4 and 0.6 were the same, possibly due to the limited incorporation of MPTMS in the mesopore. Thus, the remaining functional group precursors were attached to the external pore area, which further prevented the ordered formation of the hexagonal mesoporous structure. This postulation indicated that NRHMS-SO_3_H (0.6) provided relatively high acidity but a disordered mesoporous structure and subpar textural properties. Conversely, NRHMS-SO_3_H (0.4) provided comparable acidity to NRHMS-SO_3_H (0.6), while retaining decent textural properties, further affecting its catalytic activity.

### 3.2. The Esterification of LA with Ethanol over Various Solid Acid Catalysts

**Figure 8:**
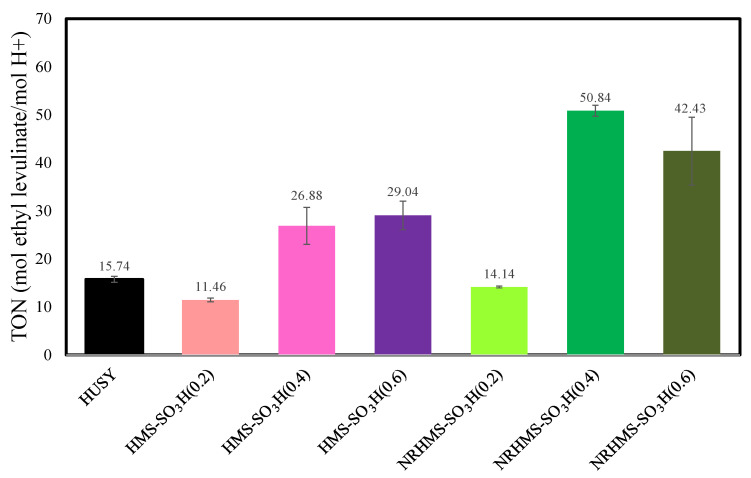
Turnover number (TON) of different solid acid catalysts used in the LA esterification with ethanol (reaction condition: Catalyst loading, 2.5 wt.% based on LA weight; LA:ethanol molar ratio, 1:5; reaction temperature, 80 °C; reaction time, 5 h; TON was calculated from moles of total ester formed divided by the total number of acid sites determined via acid–base titration).

HUSY zeolite, HMS-SO_3_H, and NRHMS-SO_3_H were comparatively tested for the esterification of LA with ethanol. As shown in Figure 8, HUSY zeolite produced a turnover number (TON) of 15.74. Most of the synthesized mesoporous catalysts provided relatively high activity compared to HUSY, due to its microporous network, thereby limiting the diffusion of reactants to the active sites. Thus, intermediates and transition states in the esterification of LA possibly avoided accommodating inside the HUSY structure, and the reaction occurred at the external surface [12].

HMS-SO_3_H(0.2) and NRHMS-SO_3_H(0.2) exhibited low TONs of 13.63 and 15.02, respectively. Despite relatively high surface area and pore size, a low amount of active functional groups may not be conducive to efficient LA esterification [46]. Ogino et al. [15] proposed the effect of surface functional groups on the performance of solid acid catalysts, wherein the remaining hydroxyl groups interacted with γ-keto groups of LA, thus concentrating the reactant molecules on the catalyst surface and facilitating the esterification. This study addressed the importance of suitable acid site density, essential for outstanding catalytic activity. For HMS-SO_3_H (0.4) and NRHMS-SO_3_H (0.4), an increase in the acid site density improved their TON values to 24.99 and 48.13, respectively. However, using a high MPTMS/TEOS ratio of 0.6 reduced TON. The considerably high density of acid sites on HMS-SO_3_H (0.6) and NRHMS-SO_3_H (0.6) hampered the product yield due to the increased water affinity on the catalyst surface and decreased surface area, promoting reversed esterification [47,48]. Moreover, for all MPTMS loading levels, NRHMS-SO_3_H showed higher activity than the HMS-SO_3_H counterparts, suggesting that the rubber moieties in the nanocomposite catalysts increased the surface hydrophobicity, preventing the adsorption of water molecules, thereby retarding the ester hydrolysis.

**Figure 9:**
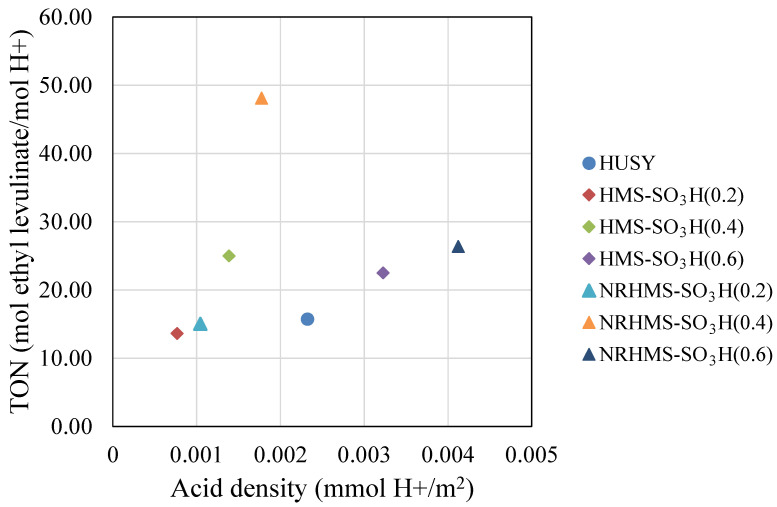
Dependence of TON on the acid site density of different solid catalysts.

TON was then plotted as a function of acid site density to reveal the major factor affecting the catalytic activity. As shown in Figure 9, the density of acid sites strongly influenced the activity of these catalysts in LA esterification. Nevertheless, the correlation curves categorized the catalysts into two groups. The catalysts with relatively low V_m, H2O_ (Table 2) exhibited TON values more sensitive to the acid site density, revealing the crucial role of hydrophobic properties of the solid catalysts in ethyl levulinate formation. From the catalyst screening study, we concluded that NRHMS-SO_3_H (0.4) was a suitable catalyst for LA esterification with ethanol due to its optimum surface hydrophobicity and acid site density.

### 3.3. Effect of Alcohol Chain Length on LA Esterification

**Figure 10:**
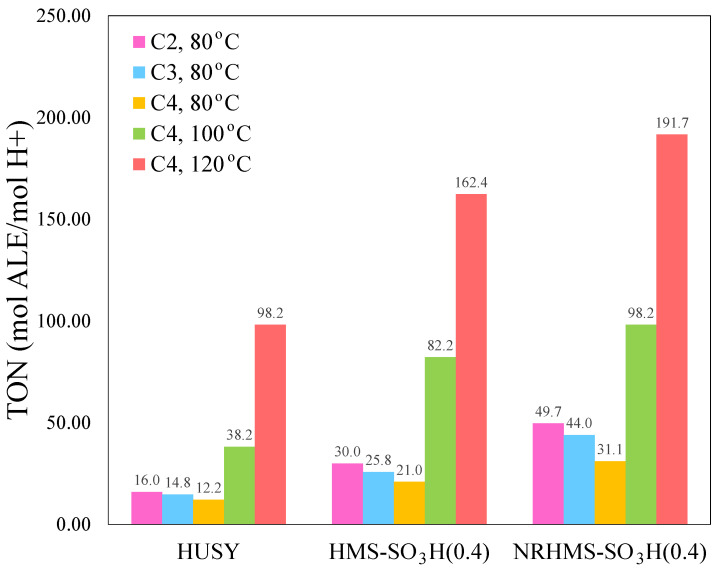
Catalytic activity of solid acids in the LA esterification with various alcohols (C2 = ethanol, C3 = *n*-propanol, and C4 = *n*-butanol) at different reaction temperatures (T = 80 °C, 100 °C, and 120 °C) (reaction condition: Catalyst loading, 2.5 wt.% based on LA weight; LA:alcohol molar ratio, 1:5; reaction time, 5 h).

Figure 10 shows the effect of the alcohol chain length (ethanol, *n*-propanol, and *n*-butanol) on the LA esterification over HUSY and mesostructured catalysts at 80 °C. The yield of corresponding esters decreased as the chain length of primary alcohols increased, attributing a decrease in the nucleophilic nature of alcohols with increased molecular size, hindering their ability to donate electron pairs to the carbonyl atom of the LA molecule [49]. This steric effect also played a crucial role in determining the TON value. The accessibility of catalytically active sites for the LA esterification must be hindered for large alcohols. As Pappu et al. [50] confirmed the mechanism of homogeneously and heterogeneously catalyzed esterification was similar to that examined by the Taft equation, the importance of these effects was validated for the heterogeneous catalyst process [51]. Regardless of the alcohol type, NRHMS-SO_3_H (0.4) still provided the highest TON, emphasizing that the hydrophobic properties of the catalyst surface could compensate for the reduced textural and acidic properties by incorporating the rubber phase into the NRHMS-SO_3_H materials.

### 3.4. Effect of Reaction Temperature on the LA Esterification

In this study (Figure 10), the reaction temperature was varied at 80 °C, 100 °C, and 120 °C using *n*-butanol as the primary alcohol, with the highest boiling point (~118 °C) minimizing the evaporative loss during the experiment. NRHMS-SO_3_H (0.4) still possessed the highest catalytic activity at all reaction temperatures. The ester product yield and TON were remarkably increased at the elevated reaction temperature, explained by the endothermic nature of esterification [46]. Additionally, the increased temperature enhanced LA miscibility in *n*-butanol and decreased the viscosity of the reactant mixture, thus facilitating the electron transfer between the reactant molecules and finally increasing the rate of esterification [52,53]. Ester hydrolysis was likely retarded at a high temperature since water (the by-product of esterification) started evaporating.

#### 3.4.1. Kinetic Study of LA Esterification

**Figure 11:**
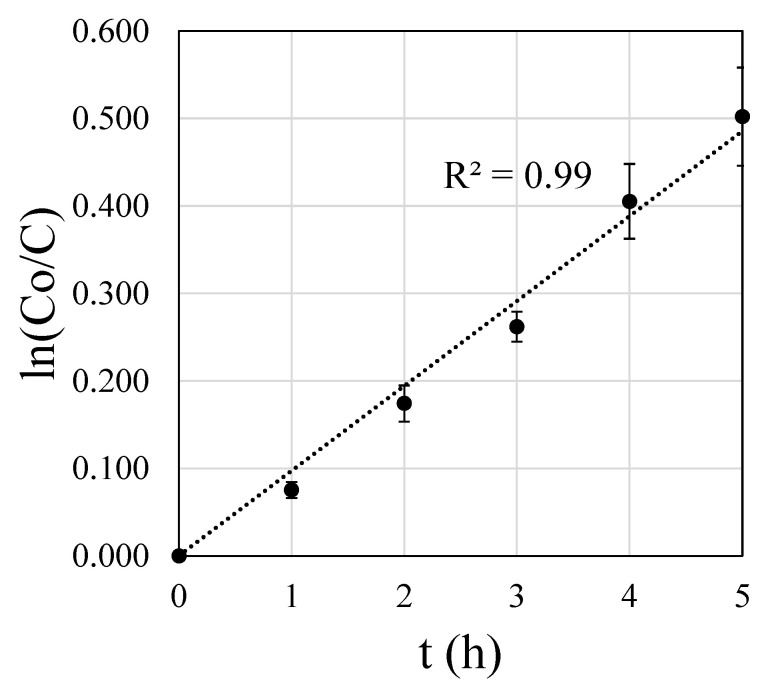
Plot of pseudo-first-order kinetic model of LA esterification with *n*-butanol over NRHMS-SO_3_H (0.4) at 120 °C.

The pseudo-first-order kinetic model was used due to excessive *n*-butanol, implying that esterification primarily depended on the LA concentration, as inferred from the linearity of the model, as presented in Figure 11. The same model was also used for the regression analysis of experimental data at different reaction temperatures, as shown in Equation (1) [54].

(1) $$\;\ln\left(\frac{C_{\mathrm{LA}.0}}{C_{\mathrm{LA}.t}}\right)=\mathrm{kt}$$

where C_LA.0_ represents the initial concentration of LA (mmol/h), C_LA.t_ represents the concentration of LA (mmol/h) at any given time, t (h), and k is the rate constant (h^−1^).

The pseudo-first-order kinetic rate constant, obtained from linear regression, is summarized in Table 3. The rate constant and the ester yield increased upon elevating the reaction temperature. In addition, the presence of a solid acid catalyst, NRHMS-SO_3_H (0.4), would significantly increase the rate constant.

Under mild conditions (T = 80 °C), the presence of catalysts noticeably affected the product yield, as presented in Figure S4. (SM). Without any catalyst, the LA molecule required more time to esterify with alcohols. For HUSY, the esterification was slightly promoted due to its microporous network, limiting the diffusion process. An increased temperature enhanced the reaction rate over HUSY since the diffusion of reactant molecules into its microporosity was promoted. In the case of HMS-SO_3_H (0.4), the dependence of the reaction rate on time was similar to HUSY. It was explained by the hydrophobicity of the HMS-SO_3_H (0.4) surface on which the accessibility of reactants to active sites was limited due to water adsorption. NRHMS-SO_3_H (0.4) prominently enhanced the LA esterification because of its suitable physicochemical properties, which effectively converted the acid reactant into an ester product after the first hour of reaction.

#### 3.4.2. Activation Energy of LA Esterification with *n*-Butanol

**Figure 12:**
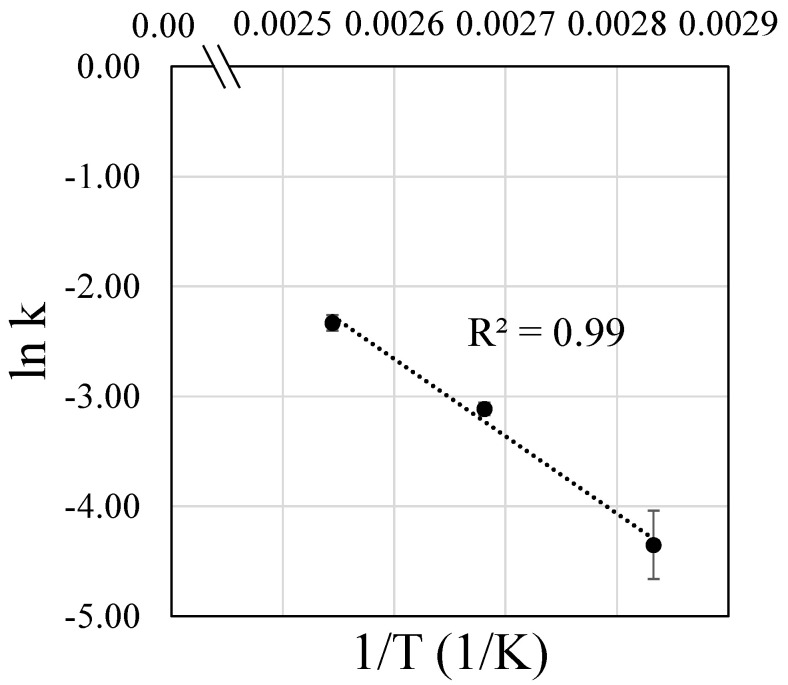
Arrhenius plot of pseudo-first-order reaction of LA esterification with *n*-butanol over NRHMS-SO_3_H (0.4).

The activation energy of LA esterification with *n*-butanol, calculated from the experimental data, subjected to the Arrhenius equation (Figure 12), is summarized in Table 4. The activation energy of LA esterification without a catalyst was 71.3 ± 5.0 kJ/mol. HUSY insignificantly decreased the activation energy to 66.2 ± 8.4 kJ/mol, while the activation energy of LA esterification over HMS-SO_3_H (0.4) was 65.2 ± 9.7 kJ/mol. As mentioned above, this was a result of unsuitable physicochemical properties, which traded off between porosity and hydrophobicity. The presence of NRHMS-SO_3_H (0.4) remarkably decreased the activation energy to 58.2 ± 7.1 kJ/mol, lower than that reported by Escobar et al. (71.1 kJ/mol) [55], which used nano core-shell magnetic materials based on the Keggin-heteropolyacids catalyst, possibly due to different reaction conditions and the esterification mechanism.

## 4. Conclusions

NRHMS-SO_3_H was successfully prepared via an in situ sol-gel method and acted as the potential solid acid catalyst for LA esterification with various alcohols. The incorporation of NR provided a hydrophobic surface, while the nanocomposite structure maintained the mesoporous framework and imparted surface functionality. The functional groups were uniformly distributed over the catalyst structure due to functionalization via the co-condensation approach. However, the high loading of MPTMS significantly diminished the physicochemical properties of materials. Among the three solid acid catalysts, NRHMS-SO_3_H (0.4) exhibited the highest catalytic activity in LA esterification with alcohols due to the synergistic effect of its physicochemical properties, acid site density, and hydrophobicity, effectively enhancing the reaction kinetics. Upon increasing the carbon chain length of the primary alcohols, catalytic activity decreased, whereas higher reaction temperatures considerably increased the ALE yield. The reaction can be described by the pseudo-first-order kinetic model. The activation energy of LA esterification with *n*-butanol of NRHMS-SO_3_H (0.4) was the lowest among all catalysts at 58.2 ± 7.1 kJ/mol, which further enhanced its catalytic performance.

## Figures and Tables

**Table 1:** Chemical composition and acidity comparison of HMS-SO_3_H and NRHMS-SO_3_H.

Sample	SiO_2_ Content ^a^ (wt.%)	S Content ^b^ (wt.%)	Theoretical Acidity ^c^ (mmol/g)	Total Acidity ^d^ (mmol/g)	Sulfur Species ^e^ (mmol/g)	
					−SH	−SO_3_H
HMS-SO_3_H (0.2)	67.8	7.8	2.45	0.69	1.87	0.58
HMS-SO_3_H (0.4)	64.4	10.3	3.22	0.79	2.36	0.85
HMS-SO_3_H (0.6)	55.4	12.2	3.80	0.90	2.64	1.16
NRHMS-SO_3_H (0.2)	62.6	7.1	2.21	0.62	1.63	0.58
NRHMS-SO_3_H (0.4)	56.0	8.6	2.68	0.71	1.95	0.74
NRHMS-SO_3_H (0.6)	54.6	11.4	3.56	0.80	2.27	1.29

^a^ Silica content determined by TGA. ^b^ Sulfur content determined by CHNS analysis. ^c^ Calculated from the total sulfur content by assuming complete oxidation of -SH to -SO_3_H. ^d^ Determined from acid–base titration. ^e^ Total sulfur content, obtained via CHNS analysis, was divided into each species using XPS analysis.

**Table 2:** Physicochemical properties of HMS-SO_3_H and NRHMS-SO_3_H series.

Sample	S_BET_^a^ (m^2^ g^−1^)	S_ext_ ^b^ (m^2^ g^−1^)	V_T_^c^ (cm^3^ g^−1^)	V_P_^d^ (cm^3^ g^−1^)	D_p_^e^ (nm)	d_100_^f^ (nm)	a_o_^g^ (nm)	W_T_^h^ (nm)	V_m, H2O_^i^ (cm^3^_STP_ g^−1^)
HMS-SO_3_H (0.2)	901	211	0.97	0.35	2.20	3.84	4.43	2.23	63.8
HMS-SO_3_H (0.4)	580	117	0.80	0.16	2.05	4.01	4.63	2.58	68.2
HMS-SO_3_H (0.6)	279	37	0.41	0.12	2.05	*n.d.*	*n.d.*	*n.d.*	72.8
NRHMS-SO_3_H (0.2)	594	177	0.81	0.17	2.15	4.01	4.63	2.48	56.7
NRHMS-SO_3_H (0.4)	400	125	0.71	0.13	2.05	4.30	4.97	2.92	58.1
NRHMS-SO_3_H (0.6)	194	47	0.36	0.07	2.05	*n.d.*	*n.d.*	*n.d.*	65.8

*n.d.*, not determined. ^a^ BET surface area. ^b^ External surface area determined from the slope of t-plot curves. ^c^ Total pore volume. ^d^ Mesopore volume determined from the interception of t-plot curves. ^e^ Pore diameter determined from BJH method. ^f^ d-spacing of (100) plane, obtained from XRD analysis. ^g^ Lattice parameter of the hexagonal unit cell. ^h^ Wall thickness determined from the subtraction between pore diameter and unit cell parameter. ^i^ Monolayer adsorbed water determined from H_2_O adsorption-desorption measurement.

**Table 3:** Alkyl levulinate esters (ALEs) yield and kinetic parameters obtained from heterogeneously catalyzed LA esterification under different reaction conditions.

Catalyst	Alcohol	Temperature (°C)	Rate of Reaction (mmol ALE/h)	k (h^−1^)
Blank	ethanol	80	0.165 ± 0.046	0.0065 ± 0.0017
	*n*-propanol	80	0.163 ± 0.023	0.0063 ± 0.0006
	*n*-butanol	80	0.150 ± 0.049	0.0052 ± 0.0008
	*n*-butanol	100	0.521 ± 0.084	0.0220 ± 0.0037
	*n*-butanol	120	1.281 ± 0.035	0.0671 ± 0.0040
HUSY	ethanol	80	0.242 ± 0.058	0.0097 ± 0.0023
	*n*-propanol	80	0.223 ± 0.052	0.0085 ± 0.0018
	*n*-butanol	80	0.184 ± 0.052	0.0072 ± 0.0021
	*n*-butanol	100	0.576 ± 0.011	0.0246 ± 0.0007
	*n*-butanol	120	1.481 ± 0.100	0.0715 ± 0.0065
HMS-SO_3_H (0.4)	ethanol	80	0.344 ± 0.151	0.0138 ± 0.0059
	*n*-propanol	80	0.295 ± 0.053	0.0117 ± 0.0019
	*n*-butanol	80	0.241 ± 0.093	0.0094 ± 0.0034
	*n*-butanol	100	0.942 ± 0.164	0.0406 ± 0.0071
	*n*-butanol	120	1.860 ± 0.269	0.0892 ± 0.0137
NRHMS-SO_3_H (0.4)	ethanol	80	0.512 ± 0.214	0.0209 ± 0.0089
	*n*-propanol	80	0.452 ± 0.157	0.0185 ± 0.0063
	*n*-butanol	80	0.320 ± 0.127	0.0129 ± 0.0033
	*n*-butanol	100	1.011 ± 0.042	0.0444 ± 0.0026
	*n*-butanol	120	1.973 ± 0.136	0.0971 ± 0.0066

Reaction condition: Catalyst loading, 2.5 wt.% based on LA weight; LA:alcohol molar ratio, 1:5; reaction time, 5 h.

**Table 4:** Activation energy (E_a_) of LA esterification with *n*-butanol over different catalysts.

Catalyst	E_a_ (kJ/mol)
Blank	71.3 ± 5.0
HUSY	66.2 ± 8.4
HMS-SO_3_H (0.4)	65.2 ± 9.7
NRHMS-SO_3_H (0.4)	58.2 ± 7.1

## Data Availability

The datasets analyzed in the current study are available from the corresponding author on reasonable request.

## Supplementary Materials

The following supporting information can be downloaded at: https://www.mdpi.com/article/10.3390/nano12040604/s1, Figure S1: Representative ATR-FTIR spectra of the synthesized materials.; Figure S2. Wide scan XPS spectrum of HMS-SO_3_H(0.2).; Figure S3. Core level high resolution (A) O1s and (B) S2p spectra of HMS-SO_3_H(0.2).; Figure S4. Wide scan XPS spectrum of HMS-SO_3_H(0.4).; Figure S5. Core level high resolution (A) O1s and (B) S2p spectra of HMS-SO_3_H(0.4).; Figure S6. Wide scan XPS spectrum of HMS-SO_3_H(0.6).; Figure S7. Core level high resolution (A) O1s and (B) S2p spectra of HMS-SO_3_H(0.6).; Figure S8. Wide scan XPS spectrum of NRHMS-SO_3_H(0.2).; Figure S9. Core level high resolution (A) O1s and (B) S2p spectra of NRHMS-SO_3_H(0.2).; Figure S10. Wide scan XPS spectrum of NRHMS-SO_3_H(0.4).; Figure S11. Core level high resolution (A) O1s and (B) S2p spectra of NRHMS-SO_3_H(0.4).; Figure S12. Wide scan XPS spectrum of NRHMS-SO_3_H(0.6).; Figure S13. Core level high resolution (A) O1s and (B) S2p spectra of NRHMS-SO_3_H(0.6).; Figure S14. Representative plot of pseudo-first order kinetic model of LA esterification with alcohols over a series of solid acid catalysts at different reaction temperature. (EtLA = ethyl levulinate, PrLA = *n*-propyl levulinate and BuLA = *n*-butyl levulinate).; Table S1. XPS binding energies and atomic percent of chemical states for HMS-SO_3_H series and NRHMS-SO_3_H series.
