# Supplementary material for: Propylsulfonic Acid-Functionalized Mesostructured Natural Rubber/Silica Nanocomposites as Promising Hydrophobic Solid Catalysts for Alkyl Levulinate Synthesis
Source: Nanomaterials (Basel). 2022 Feb 11;12(4):604. doi: 10.3390/nano12040604 (PMC8877181; doi:10.3390/nano12040604)
Supplement: Supplementary file 1 [file nanomaterials-12-00604-s001.zip › nanomaterials-1554169-supplementary.pdf]

Supplementary Material

Propylsulfonic Acid-Functionalized Mesostructured Natural Rubber/Silica Nanocomposites as Promising Hydrophobic Solid Catalysts for Alkyl Levulinate Synthesis

Supphathee Chaowamalee [1,2], Ning Yan [3] and Chawalit Ngamcharussrivichai [1,2,4,*]

1 Department of Chemical Technology, Faculty of Science, Chulalongkorn University, Bangkok 10330, Thailand; sup_7788@hotmail.com
2 Center of Excellence on Petrochemical and Materials Technology (PETROMAT), Chulalongkorn University, Bangkok 10330, Thailand
3 Department of Chemical and Biomolecular Engineering, National University of Singapore, 4 Engineering Drive 4, Singapore 117585, Singapore; ning.yan@nus.edu.sg
4 Center of Excellence in Catalysis for Bioenergy and Renewable Chemicals (CBRC), Faculty of Science, Chulalongkorn University, Bangkok 10330, Thailand
* Correspondence: chawalit.ng@chula.ac.th; Tel.: +66-2-218-7528; Fax: +66-2-255-5831

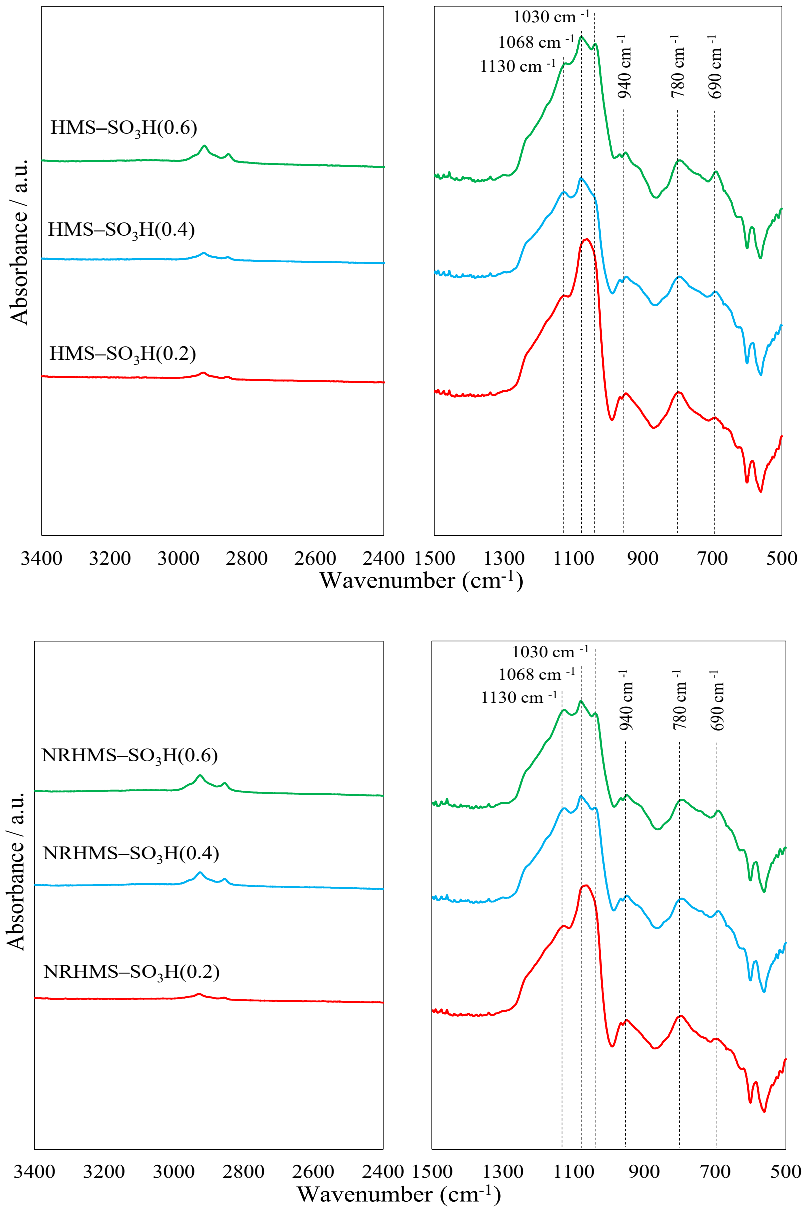

Figure S1. Representative ATR-FTIR spectra of the synthesized materials.

The Si−O stretching of silanol group was noticed at 940 cm^{-1}. The broad band was detected in the range of 1000 to 1300 cm^{-1} with a major band at 1068 cm^{-1}, representing the asymmetric Si−O−Si stretching, while another band at 780 cm^{-1} was assigned to the symmetric Si−O−Si stretching. The characteristic peaks of the functionalized group were also detected. The small band at 690 cm^{-1} represented sulfonic acid group bending vibration. The three bands overlapped in the asymmetric Si−O−Si stretching region, which became clearer at higher degree of MPTMS loading. The first two bands were observed at 1030 cm^{-1} and 1130 cm^{-1}, corresponding to the O=S=O symmetric and asymmetric stretching vibration, respectively.

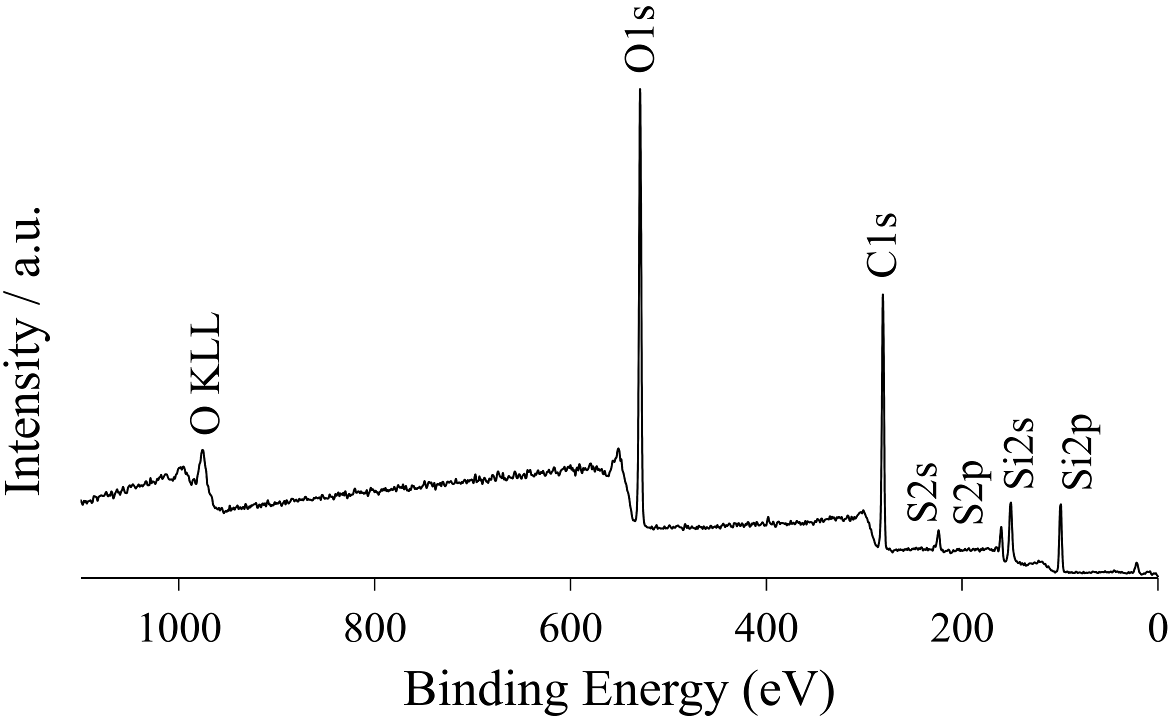

Figure S2. Wide scan XPS spectrum of HMS-SO_3H(0.2).

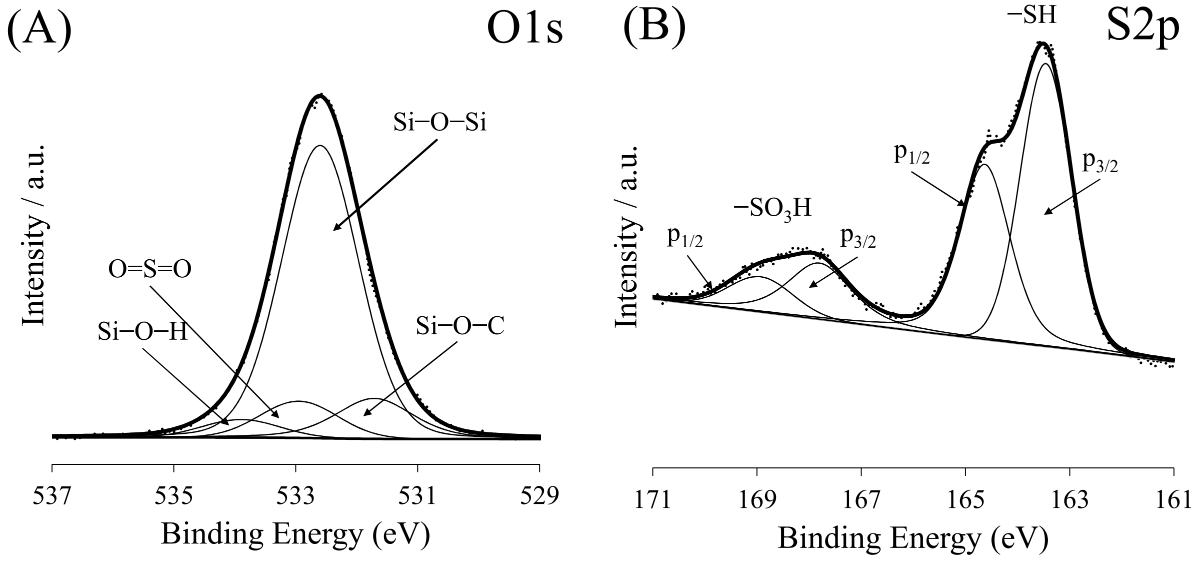

Figure S3. Core level high resolution (A) O1s and (B) S2p spectra of HMS-SO_3H(0.2).

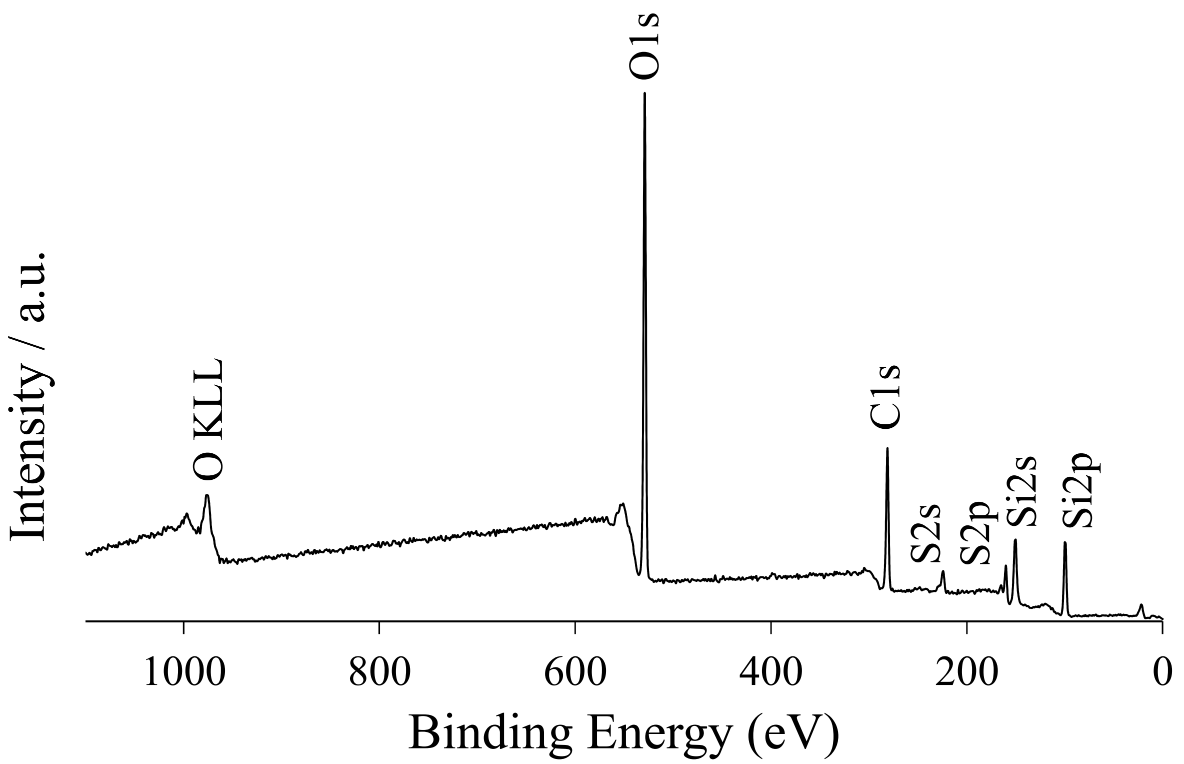

Figure S4. Wide scan XPS spectrum of HMS-SO_3H(0.4).

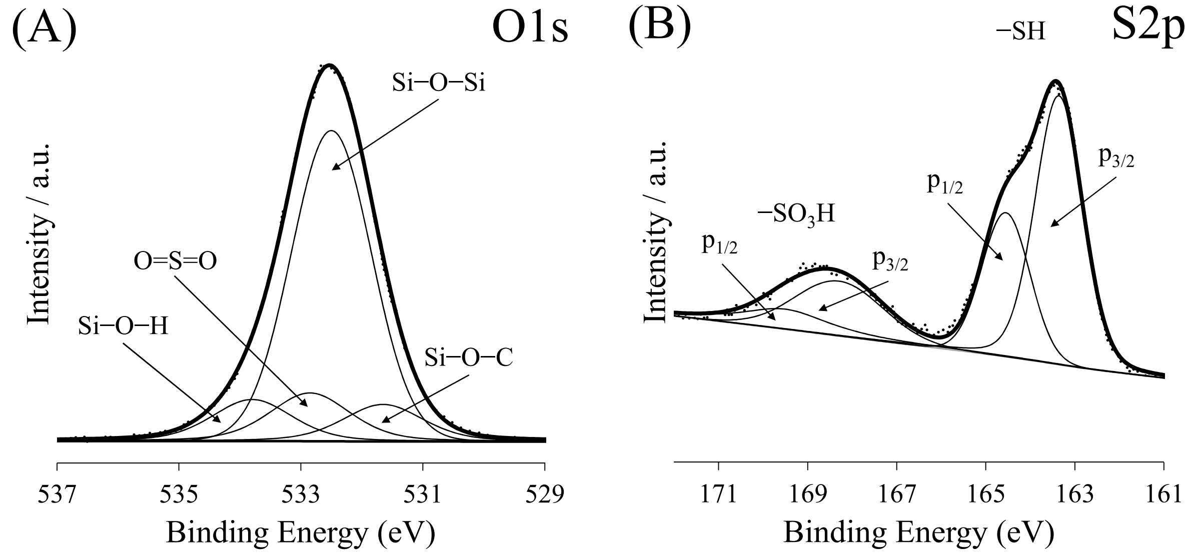

Figure S5. Core level high resolution (A) O1s and (B) S2p spectra of HMS-SO_3H(0.4).

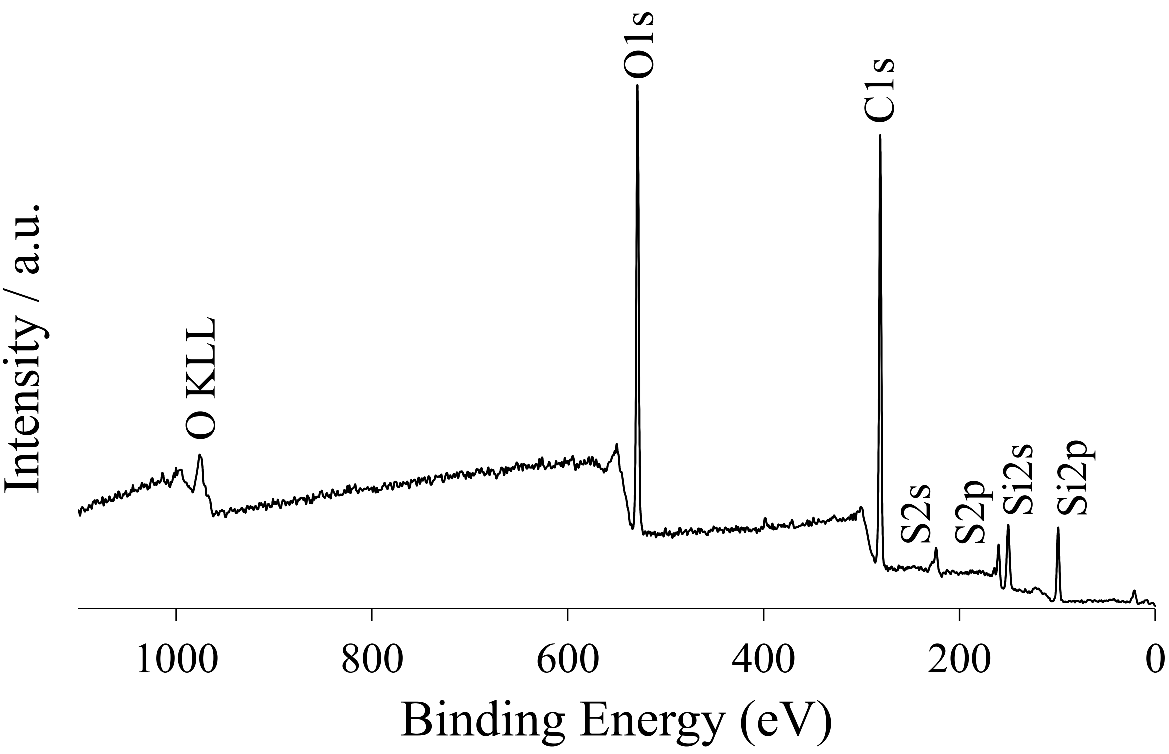

Figure S6. Wide scan XPS spectrum of HMS-SO_3H(0.6).

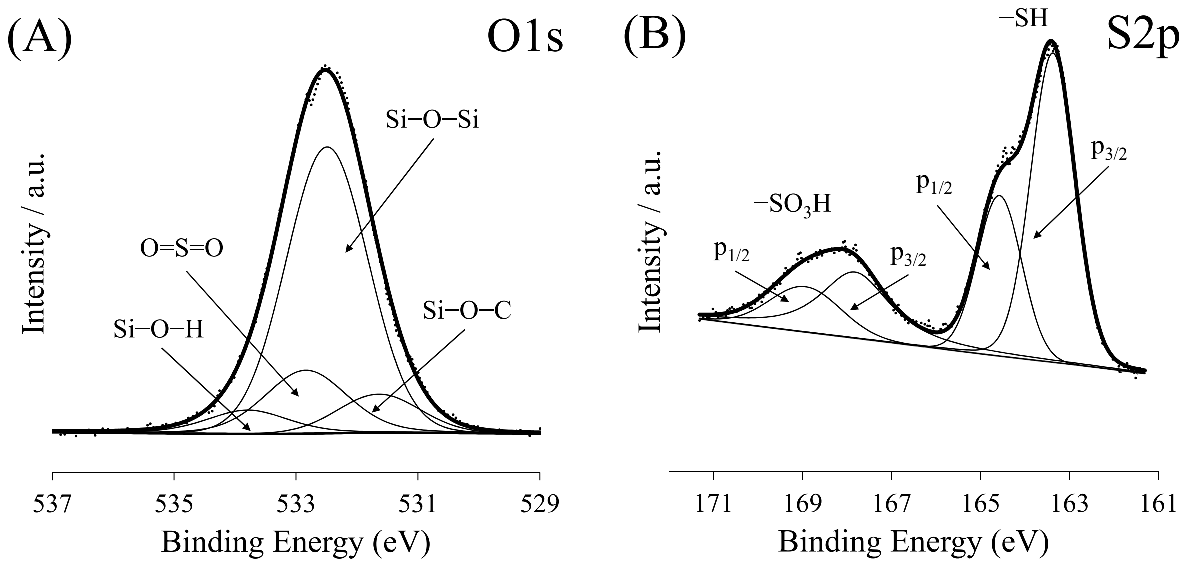

Figure S7. Core level high resolution (A) O1s and (B) S2p spectra of HMS-SO_3H(0.6).

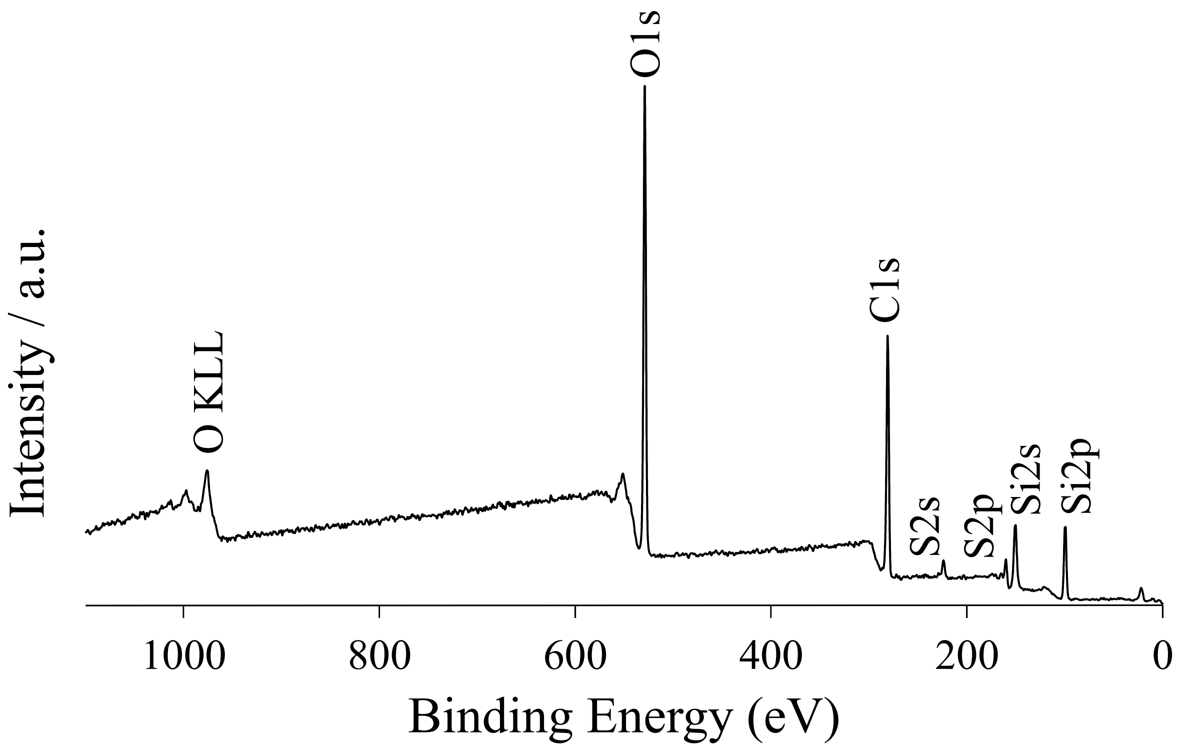

Figure S8. Wide scan XPS spectrum of NRHMS-SO_3H(0.2).

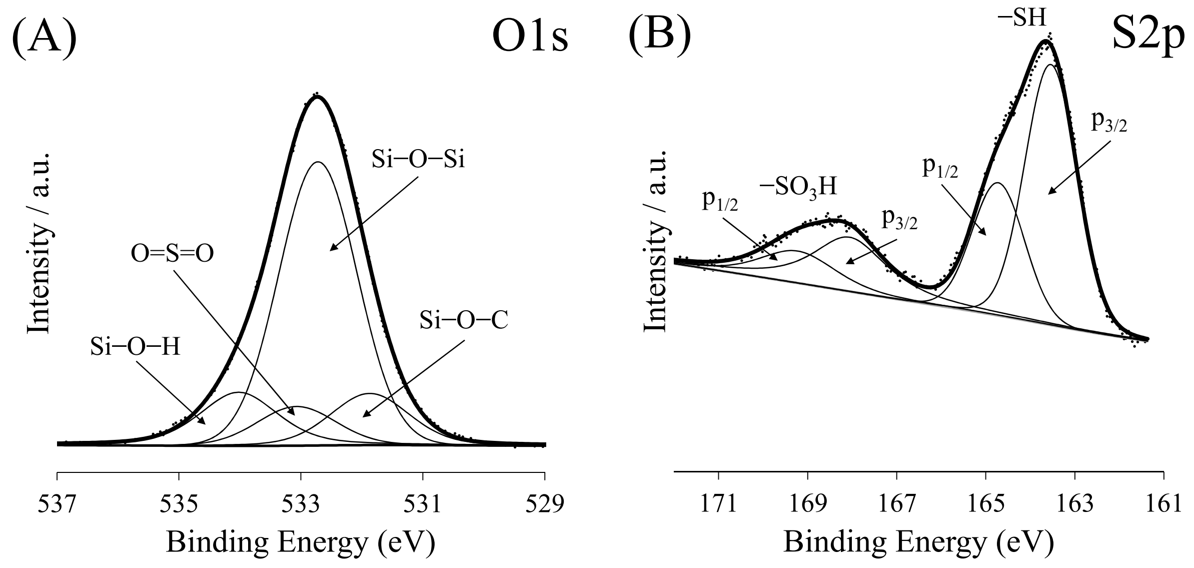

Figure S9. Core level high resolution (A) O1s and (B) S2p spectra of NRHMS-SO_3H(0.2).

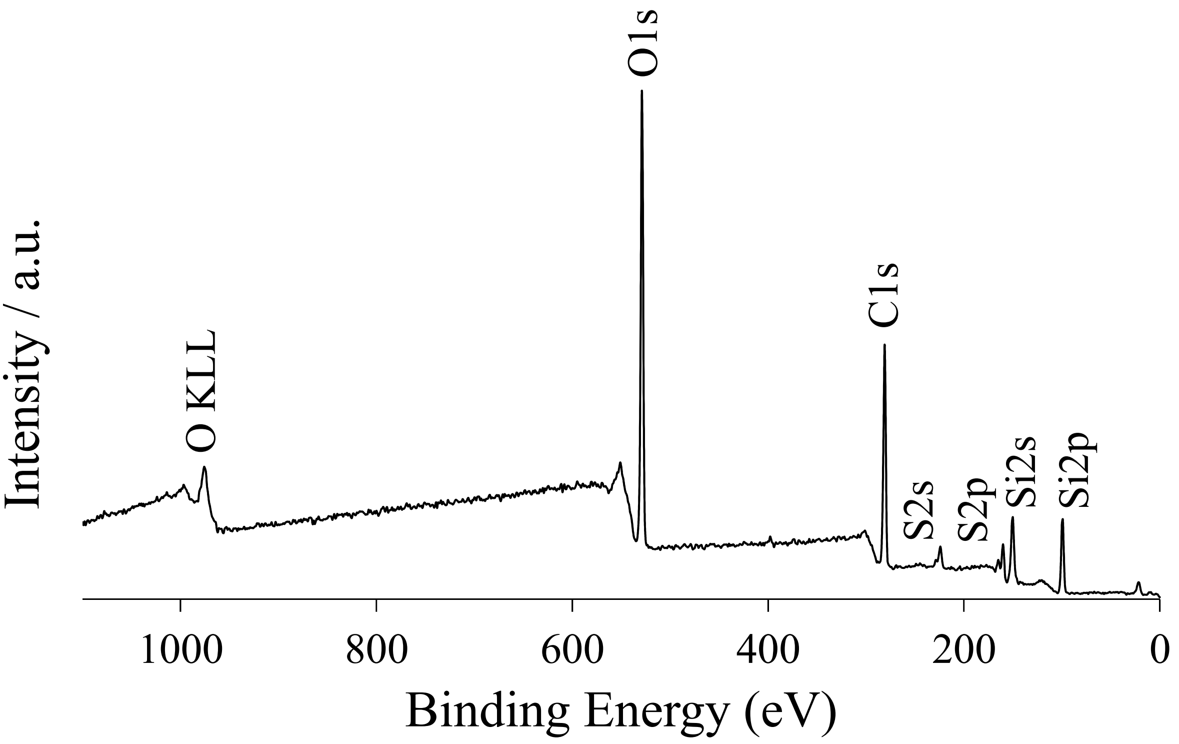

Figure S10. Wide scan XPS spectrum of NRHMS-SO_3H(0.4).

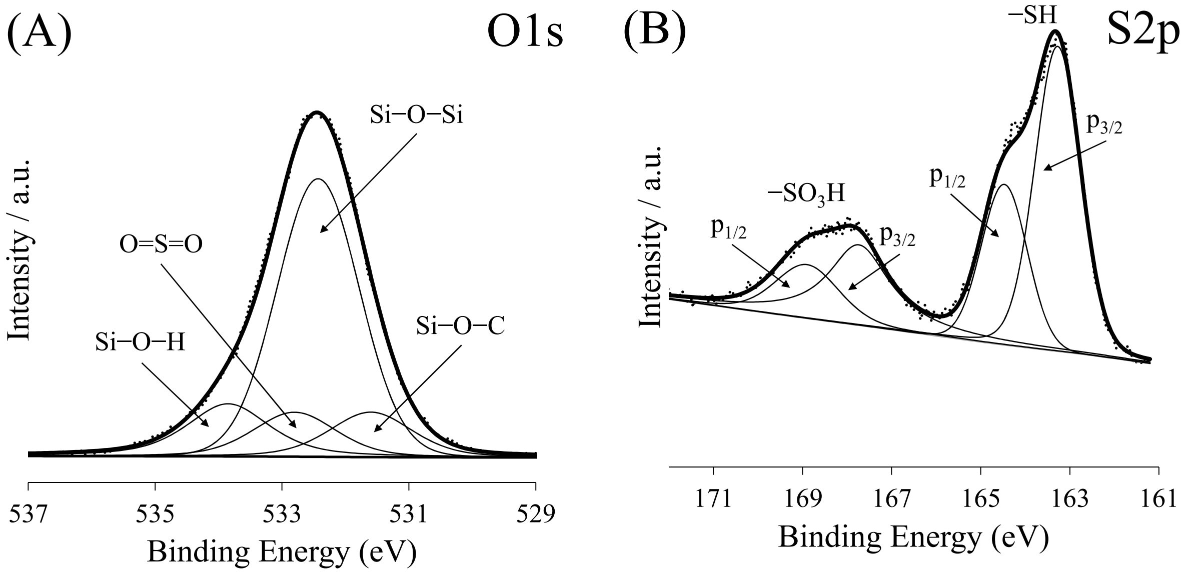

Figure S11. Core level high resolution (A) O1s and (B) S2p spectra of NRHMS-SO_3H(0.4).

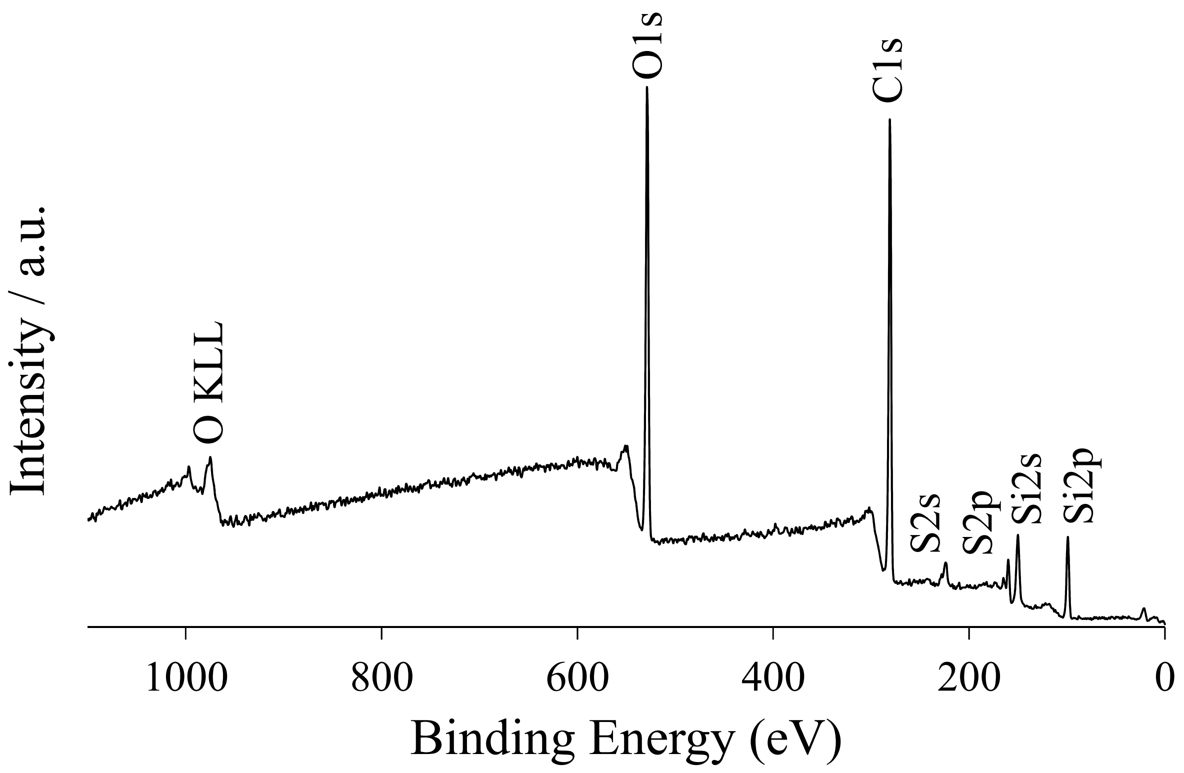

Figure S12. Wide scan XPS spectrum of NRHMS-SO_3H(0.6).

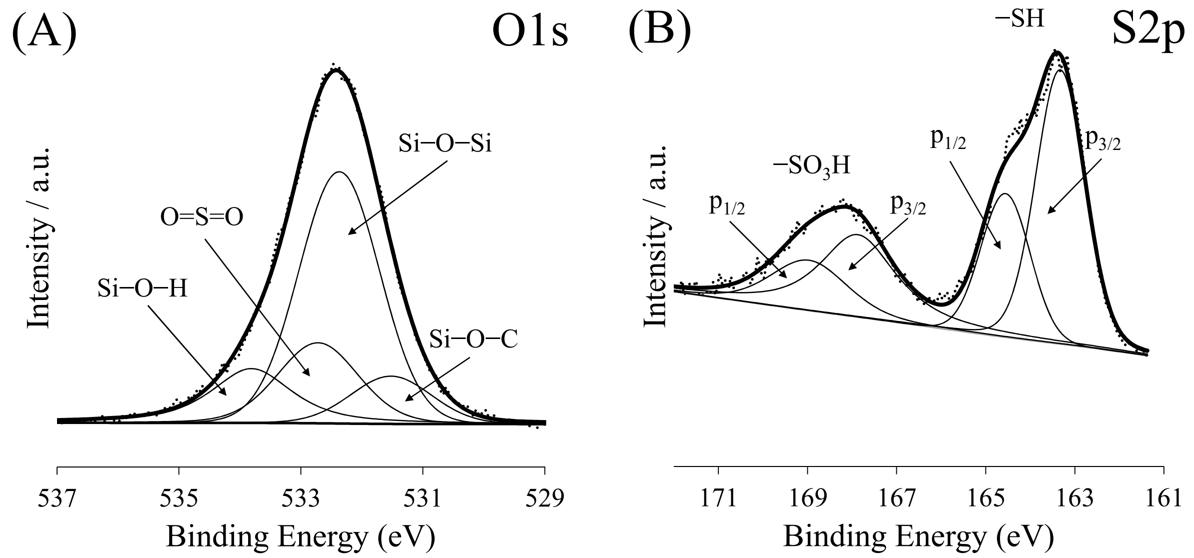

Figure S13. Core level high resolution (A) O1s and (B) S2p spectra of NRHMS-SO_3H(0.6).

Table S1. XPS binding energies and atomic percent of chemical states for HMS-SO_3H series and NRHMS-SO_3H series.

Core peak	HMS-SO_3H(0.2)		HMS-SO_3H(0.4)		HMS-SO_3H(0.6)	
	BE/eV (FWHM/eV)	%rc	BE/eV (FWHM/eV)	%rc	BE/eV (FWHM/eV)	%rc
O1s						
Si−O−C	531.8 (1.6)	5.96	531.8 (1.6)	11.74	531.8 (1.6)	15.23
Si−O−Si	532.6 (1.6)	74.57	532.6 (1.6)	63.29	532.6 (1.6)	55.60
O=S=O	533.0 (1.6)	16.88	533.0 (1.6)	19.67	533.0 (1.6)	24.16
Si−O−H	533.9 (1.6)	2.59	533.9 (1.6)	5.30	533.9 (1.6)	5.01
S2p						
−SH $p_{3/2}$	163.6 (1.2)	50.85	163.6 (1.2)	48.97	163.6 (1.2)	46.31
−SH $p_{1/2}$	164.8 (1.2)	25.43	164.8 (1.2)	24.48	164.8 (1.2)	23.15
−SO_3H $p_{3/2}$	168.0 (1.6)	15.82	168.1 (1.6)	17.70	168.1 (1.6)	20.36
−SO_3H $p_{1/2}$	169.2 (1.6)	7.91	169.3 (1.6)	8.85	169.3 (1.6)	10.18

Core peak	NRHMS-SO_3H(0.2)		NRHMS-SO_3H(0.4)		NRHMS-SO_3H(0.6)	
	BE/eV (FWHM/eV)	%rc	BE/eV (FWHM/eV)	%rc	BE/eV (FWHM/eV)	%rc
O1s						
Si−O−C	531.8 (1.6)	7.74	531.8 (1.6)	12.05	531.8 (1.6)	15.89
Si−O−Si	532.6 (1.6)	67.11	532.6 (1.6)	59.41	532.6 (1.6)	51.46
O=S=O	533.0 (1.6)	14.89	533.0 (1.6)	19.19	533.0 (1.6)	22.43
Si−O−H	533.9 (1.6)	10.26	533.9 (1.6)	9.35	534.0 (1.6)	10.22
S2p						
−SH $p_{3/2}$	163.6 (1.2)	49.06	163.6 (1.3)	48.38	163.6 (1.4)	42.58
−SH $p_{1/2}$	164.8 (1.2)	24.53	164.8 (1.3)	24.19	164.8 (1.4)	21.29
−SO_3H $p_{3/2}$	168.1 (1.6)	17.61	168.1 (1.6)	18.28	168.1 (1.9)	24.09
−SO_3H $p_{1/2}$	169.3 (1.6)	8.80	169.3 (1.6)	9.14	169.3 (1.9)	12.04

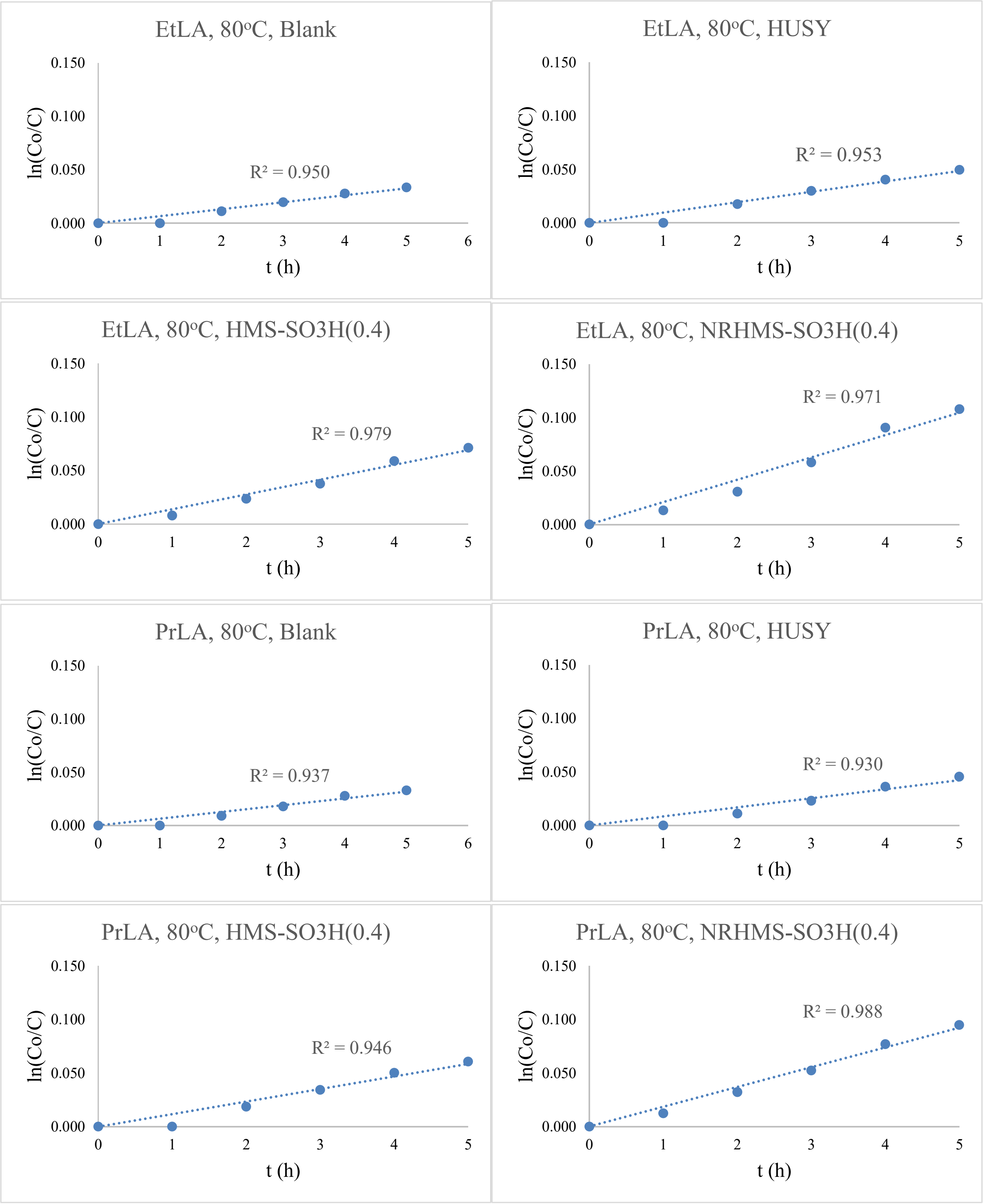
EtLA, 80ºC, Blank
ln(Co/C)
t (h)
R² = 0.950
EtLA, 80ºC, HUSY
R² = 0.953
EtLA, 80ºC, HMS-SO3H(0.4)
R² = 0.979
EtLA, 80ºC, NRHMS-SO3H(0.4)
R² = 0.971
PrLA, 80ºC, Blank
R² = 0.937
PrLA, 80ºC, HUSY
R² = 0.930
PrLA, 80ºC, HMS-SO3H(0.4)
R² = 0.946
PrLA, 80ºC, NRHMS-SO3H(0.4)
R² = 0.988

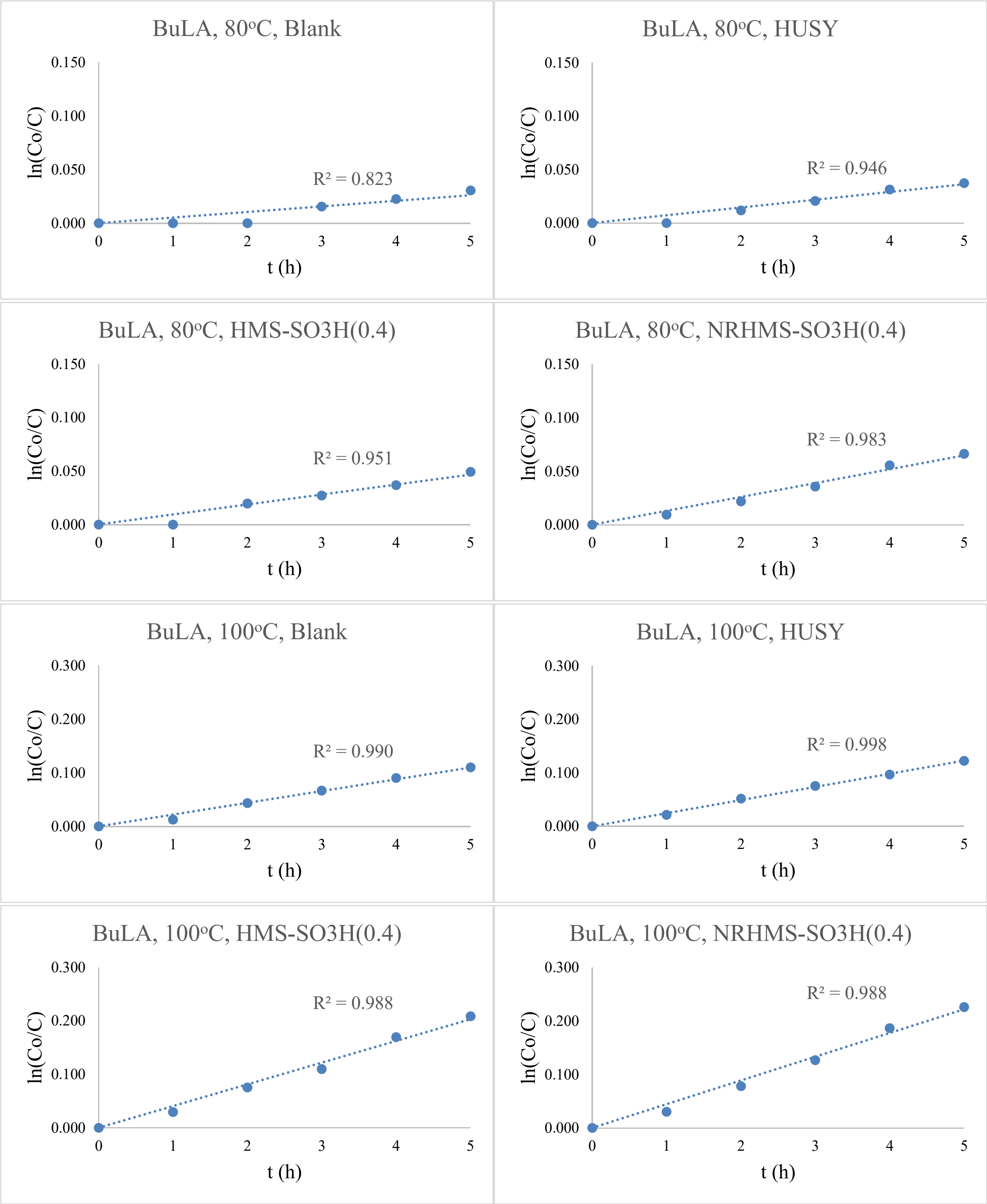
BuLA, 80ºC, Blank
ln(Co/C)
0.150
0.100
0.050
0.000
0
1
2
3
4
5
t (h)
R² = 0.823
BuLA, 80ºC, HUSY
ln(Co/C)
0.150
0.100
0.050
0.000
0
1
2
3
4
5
t (h)
R² = 0.946
BuLA, 80ºC, HMS-SO3H(0.4)
ln(Co/C)
0.150
0.100
0.050
0.000
0
1
2
3
4
5
t (h)
R² = 0.951
BuLA, 80ºC, NRHMS-SO3H(0.4)
ln(Co/C)
0.150
0.100
0.050
0.000
0
1
2
3
4
5
t (h)
R² = 0.983
BuLA, 100ºC, Blank
ln(Co/C)
0.300
0.200
0.100
0.000
0
1
2
3
4
5
t (h)
R² = 0.990
BuLA, 100ºC, HUSY
ln(Co/C)
0.300
0.200
0.100
0.000
0
1
2
3
4
5
t (h)
R² = 0.998
BuLA, 100ºC, HMS-SO3H(0.4)
ln(Co/C)
0.300
0.200
0.100
0.000
0
1
2
3
4
5
t (h)
R² = 0.988
BuLA, 100ºC, NRHMS-SO3H(0.4)
ln(Co/C)
0.300
0.200
0.100
0.000
0
1
2
3
4
5
t (h)
R² = 0.988

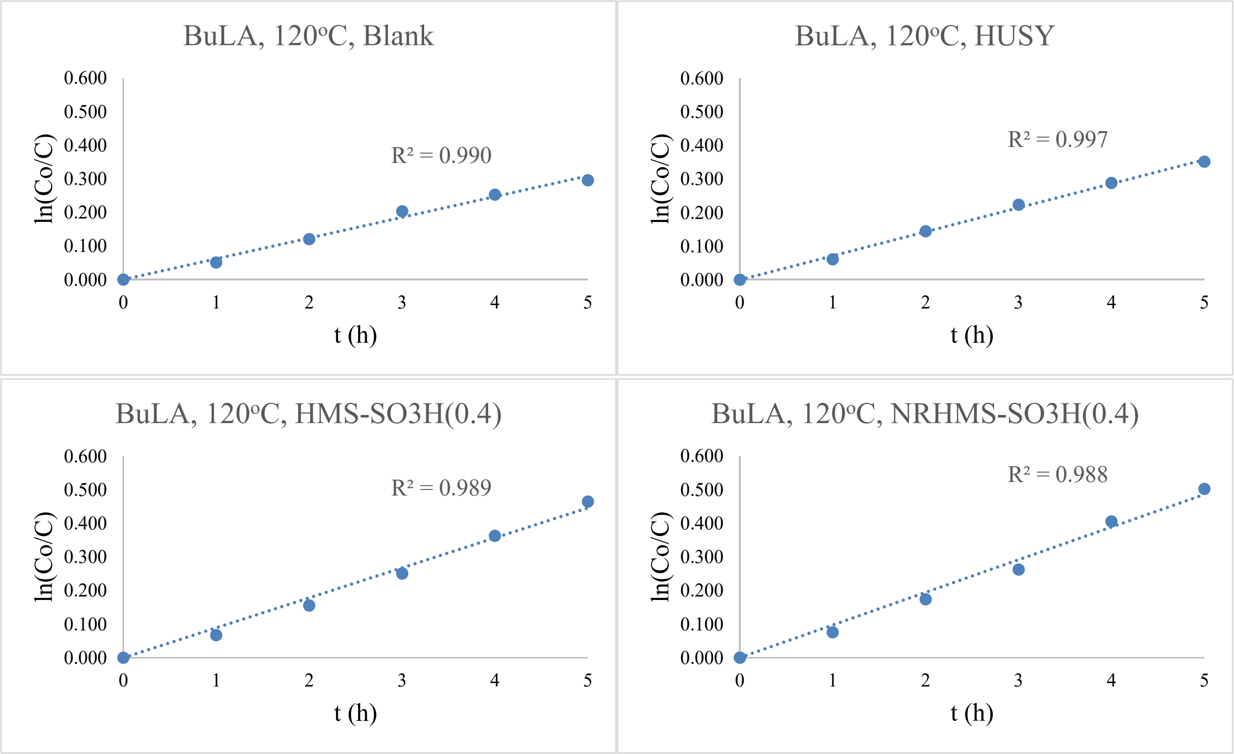

Figure S14. Representative plot of pseudo-first order kinetic model of LA esterification with alcohols over a series of solid acid catalysts at different reaction temperature. (EtLA = ethyl levulinate, PrLA = *n*-propyl levulinate and BuLA = *n*-butyl levulinate)

The total acidity was calculated from Eq. (ii).

$$N = \left(\frac{C \times V}{1000 \times W}\right) \quad , \qquad \text{(ii)}$$

where N is the acidity (mmol/g), C is the concentration of NaOH aqueous solution, V is the volume of NaOH aqueous solution used in titration (mL), and W is the sample weight (g).
